# Interleukin-7 receptor signaling is crucial for enhancer-dependent TCRδ germline transcription mediated through STAT5 recruitment

**DOI:** 10.3389/fimmu.2022.943510

**Published:** 2022-08-19

**Authors:** Alonso Rodríguez-Caparrós, Shizue Tani-ichi, Áurea Casal, Jennifer López-Ros, Carlos Suñé, Koichi Ikuta, Cristina Hernández-Munain

**Affiliations:** ^1^ Institute of Parasitology and Biomedicine “López-Neyra”- Spanish Scientific Research Council (IPBLN-CSIC), Technological Park of Health Sciences (PTS), Granada, Spain; ^2^ Laboratory of Immune Regulation, Department of Virus Research, Institute for Life and Medical Sciences, Kyoto University, Kyoto, Japan

**Keywords:** enhancer, transcription, T-cell receptor, T-cell development, V(D)J recombination, IL-7, STAT5, γδ T cells

## Abstract

γδ T cells play important roles in immune responses by rapidly producing large quantities of cytokines. Recently, γδ T cells have been found to be involved in tissue homeostatic regulation, playing roles in thermogenesis, bone regeneration and synaptic plasticity. Nonetheless, the mechanisms involved in γδ T-cell development, especially the regulation of TCRδ gene transcription, have not yet been clarified. Previous studies have established that NOTCH1 signaling plays an important role in the *Tcrg* and *Tcrd* germline transcriptional regulation induced by enhancer activation, which is mediated through the recruitment of RUNX1 and MYB. In addition, interleukin-7 signaling has been shown to be required for *Tcrg* germline transcription, VγJγ rearrangement and γδ T-lymphocyte generation as well as for promoting T-cell survival. In this study, we discovered that interleukin-7 is required for the activation of enhancer-dependent *Tcrd* germline transcription during thymocyte development. These results indicate that the activation of both *Tcrg* and *Tcrd* enhancers during γδ T-cell development in the thymus depends on the same NOTCH1- and interleukin-7-mediated signaling pathways. Understanding the regulation of the *Tcrd* enhancer during thymocyte development might lead to a better understanding of the enhancer-dependent mechanisms involved in the genomic instability and chromosomal translocations that cause leukemia.

## Graphical Abstract

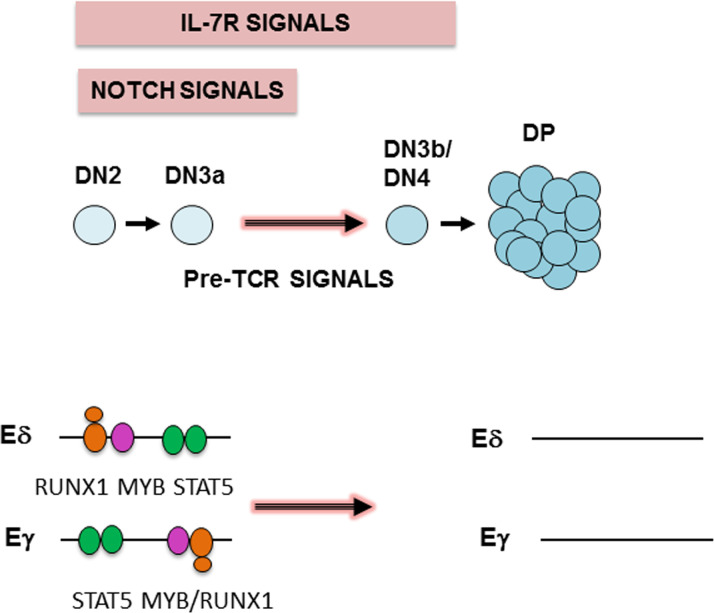

## Introduction

γδ T cells constitute a minor T-cell population (1-10% of all T lymphocytes) compared with canonical αβ T cells (1). In addition to blood and secondary immune organs, where αβ T cells reside, γδ T cells accumulate in the gut mucosa, lung, skin, uterus, adipose tissue, meninges, liver and peritoneal cavity, playing important roles in the initiation and propagation of immune responses. During antigen recognition, γδ T cells express a T-cell receptor (TCR), TCRγδ, which can specifically respond to a variety of ligands, including nonpeptidic antigens, such as phosphoantigens and lipids that are not presented by major histocompatibility complex molecules, and peptides presented by the major histocompatibility complex (2, 3). In addition, γδ T-cell immune functions include (i) rapid production of large quantities of cytokines, (ii) killing of infected and tumor cells in a manner similar to natural killer cells, (iii) elimination of bacteria and other particles, and (iv) antigen presentation (1). Due to their innate and adaptive properties that enable them to robustly kill a wide range of tumor or infected cells, ability to present peptide antigens to αβ T cells, and major histocompatibility complex-independent antigen recognition, increased interest has recently been directed to their potential use in novel immunotherapies (2). In addition, important roles played by γδ T cells, including their functions in thermogenesis, bone regeneration, and synaptic plasticity, have been identified in tissue homeostasis (4–8). Despite the growing interest in these cells, the mechanism by which TCRδ gene transcription is regulated during γδ T-cell generation has not yet been clarified.

During development in the thymus, T-cell precursors transition through a series of stages in which CD4 and CD8 are differentially expressed; these intermediates include CD4^-^CD8^-^ double-negative (DN), CD4^+^CD8^+^ double-positive (DP), and CD4^+^ or CD8^+^ single-positive (SP) thymocytes (9). Four DN populations, DN1 to DN4, are distinguished by the expression of CD25 and CD44; DN2 and DN3 thymocytes can be further classified into DN2a and DN2b and DN3a and DN3b, based on the expression of CD117 and CD27, respectively (9, 10).

Ordered expression of TCRγδ and TCRαβ during thymocyte development is highly controlled to ensure the correct development of γδ and αβ T cells. TCRγ and TCRδ chains are simultaneously expressed in DN2b and DN3a thymocytes to generate TCRγδ. The TCRβ chain is expressed in DN3a thymocytes with an invariable pre-Tα chain, resulting in a TCR precursor known as pre-TCR, which induces cell proliferation, CD4 and CD8 expression leading to DP thymocyte generation, and TCRα chain expression. TCRα and TCRβ chains are then simultaneously expressed in DP and SP thymocytes to form TCRαβ. Therefore, γδ T cells arise from DN2b and DN3a thymocytes as a result of TCRγδ expression, whereas αβ T cells are derived from DP thymocytes as a result of TCRαβ expression. Because TCRγ, TCRδ and TCRβ rearrangements occur in bipotent αβ/γδ T-cell precursors, the final outcomes derived from these events have an unquestionable impact on the ultimate T-cell fate (αβ vs. γδ T-cell), which is regulated by an instructive mechanism based on the stronger signaling of TCRγδ than that mediated by the pre-TCR (11–13). Interestingly, pre-TCR signaling not only induces the expression of the TCRα chain but also induces the termination of TCRγ and TCRδ chain expression (14–16). Therefore, the exact control of the expression of these chains is crucial for the normal assembly of functional TCRs in thymocytes and the generation of γδ and αβ T cells (9, 17).

The ordered expression of the different TCR chains during thymocyte development depends on the specific regulation of enhancer-dependent germline transcription and V(D)J recombination at each individual TCR gene (9). These genes exist in two different conformations, unrearranged and rearranged, with a correctly rearranged configuration required for the expression of a functional chain (9). To pass from an unrearranged to a rearranged configuration, the enhancers present within the TCR genes play a critical role by triggering noncoding germline transcription initiated at the D and J gene segment promoters to promote accessibility of RAG proteins to the D-J region (18–20). V(D)J recombination-deficient mice, such as RAG-deficient mice, have a total block at the DN3a stage due to their inability to rearrange and express any of their TCR chains, as CD27 expression is dependent on intracellular TCRβ expression (10, 21). After rearrangement, transcription at the rearranged TCR genes depends on enhancer-dependent activation of the recombined V gene segment.

Expression of the TCRγ and TCRδ chains depends on the activity of their respective transcriptional enhancers, Eγ and Eδ, which activate germline transcription of their unrearranged respective gene and subsequent recombination in DN2b to DN3a thymocytes (22, 23). Successful VγJγ and VδDδJδ rearrangements (**Figure S1**) permit the expression of TCRγδ in these cells, which drives thymocyte differentiation into γδ T lymphocytes (10). Because *Tcrg*, *Tcrd*, and *Tcrb* germline transcription and recombination occur before TCRγδ or pre-TCR expression in bipotent αβ/γδ T-cell precursors, these events are not directly involved in αβ vs. γδ T-lineage determination, which depends on the expression of TCRγδ and pre-TCR (11–13). In DP thymocytes, *Tcrg* and *Tcrd* transcription is inactivated by pre-TCR signaling (14–16, 23).

Signaling mediated through NOTCH1 and interleukin-7 (IL-7) receptor (IL-7R) is essential for the generation of T cells (24). NOTCH1 signaling is indispensable for T-cell commitment at the DN2a thymocyte stage, and IL-7R signaling is required for thymocyte survival, proliferation and maturation and ultimately the generation of γδ T cells (25–28). Interestingly, the NOTCH1 and IL-7R signaling pathways constitute part of a transcriptional regulatory axis, in which IL-7Rα expression depends on NOTCH1 signaling (29–31). These signals are very strong in thymocytes from DN1 through the DN3a stages, decreasing abruptly during the transition to the DN3b thymocyte stage and in DP thymocytes due to inhibited NOTCH1 expression and subsequent reduction in IL-7Rα expression as a consequence of pre-TCR signaling (10, 28, 32).

Previous experiments with DN3a thymocytes demonstrated that the activity of Eγ and Eδ measured by their ability to activate *Tcrg* and *Tcrd* germline transcription is induced by NOTCH1-dependent recruitment of RUNX1 and MYB (14, 16, 33–37); these factors are dissociated in DP thymocytes because of pre-TCR signaling-dependent inhibition of *Notch1* expression, indicating a molecular mechanism of *Tcrg* and *Tcrd* silencing during thymocyte development (14). Hence, NOTCH1 plays an important role in enhancer-dependent *Tcrg* and *Tcrd* germline transcription and TCRγδ expression during thymocyte development and thus in the generation of γδ T cells. Interestingly, IL-7R signaling is required for *Tcrg* germline transcription and VγJγ rearrangement (38–43), explaining the absence of γδ T lymphocytes in *Il7ra* ^-/-^ and *Il7* ^-/-^ mice. IL-7R-dependent recruitment of STAT5 to *Tcrg* enhancers and promoters is essential for activating the noncoding germline transcription that triggers VγJγ recombination (16, 44–47). STAT5 binding to Eγ is lost in DP thymocytes because IL-7R signaling is terminated, constituting an additional mechanism of *Tcrg* silencing (16). In mature T cells, IL-7R signaling is also necessary for transcription of the rearranged *Tcrg* (48).

Hence, both IL-7R-dependent STAT5 and NOTCH1-dependent RUNX1 and MYB dissociation from Eγ cause *Tcrg* silencing in DP thymocytes (14, 16). Based on the parallel regulation of Eγ and Eδ by the NOTCH1/RUNX1 and MYB pathways in the regulation of *Tcrg* and *Tcrd* germline transcription during thymocyte development (14), we hypothesize that Eδ also depends on the IL-7R/STAT5 pathway in DN3a thymocytes, similar to Eγ. Our results demonstrate that IL-7R/STAT5 signaling is crucial for Eδ-dependent *Tcrd* germline transcription. These data indicate that Eδ and Eγ are identically regulated through the same signaling pathways mediated by NOTCH1/RUNX1 and MYB and IL-7R/STAT5 in DN3a thymocytes, revealing indistinguishable mechanisms for expressing and silencing enhancer-dependent *Tcrg* and *Tcrd* germline transcripts during thymocyte development.

## Materials and methods

### Mice

*Rag2* ^-/-^and *Il7ra* ^-/-^ mice have been described previously (27, 49). Three- to eight-week-old *Rag2* ^-/-^and *Rag2* ^-/-^ x *Il7ra* ^-/-^ mice were used in this study. The animals were housed under pathogen-free conditions in the Animal Experimentation Unit at the IPBLN-CSIC in Granada, Spain, or the Institute for Frontier Life and Medical Sciences Resources in Kyoto, Japan. All animal work followed protocols approved by the CSIC and Andalusia Government Ethical Committees or the Kyoto University Animal Care and Use Committee.

### Cells and *in vitro* stimulations, inhibitions and viral transduction

SCID.adh cells have been previously described (50). The cells used in this study were from the original parental cells, which are mostly committed to the T-cell lineage (51, 52). They were cultured in RPMI 1640 medium with 10% fetal calf serum, sodium pyruvate, nonessential amino acids, glutamine, penicillin/streptomycin, and 50 μM 2-mercaptoethanol. Jurkat-green fluorescent protein (GFP)- and Jurkat-IL7Rα-GFP-expressing cells have been previously described (53). These cells were cultured in RPMI 1640 medium with 10% fetal calf serum, glutamine, and penicillin/streptomycin. SCID.adh cells (1 x 10^5^ cells/mL) and Jurkat-GFP and Jurkat-IL7Rα-GFP cells (5 x 10^5^ cells/mL) were stimulated in culture with 10 ng/mL murine recombinant IL-7 (Peprotech) for 30 minutes to 48 hours, as indicated. SCID.adh cells (1 x 10^5^ cells/mL) were incubated with 20 ng/mL phorbol acetate myristate and 0.5 μg/mL ionomycin (Sigma–Aldrich, Merck) or 16 μM γ-secretase inhibitor 7(B-(-(3,5-difluorophenyl)-1-alanyl)-s-phenyl-glycine t-butyl ester) (DAPT) (Selleckchem) for 24 hours. Viral transduction of SCID.adh cells with MigR retroviral plasmids was previously described (14, 54).

### Quantitative reverse transcription polymerase chain reaction 

To analyze transcription in SCID.adh cells, total RNA was obtained with peqGOLD TriFast (Peqlab) or Trifast (VWR). For RT–qPCR and the analysis of enhancer RNA (eRNA) transcripts in SCID.adh cells, genomic DNA-free RNA was obtained using Nucleospin plus columns (Macherey Nagel), and contaminating genomic DNA was eliminated by treatment with RNAse-free DNaseI (2270A, Takara) in the presence of an RNase inhibitor (2313A, Takara) for 1 hour at 37°C, followed by two consecutive phenol/chloroform extraction steps (Amresco/Merck). The DNase I treatment and extraction steps were repeated, and RNA was ultimately precipitated by adding ethanol to a final concentration of 70% with RNase-free glycogen as the carrier. The presence of genomic DNA contamination was determined by quantitative PCR (qPCR) using the Eγ4 primers used in quantitative chromatin immunoprecipitation (qChIP) experiments. cDNA was obtained from 500 ng of total RNA with PrimeScript RT master mix (RR036, Takara) and dissolved in 100 μL with Milli-Q water. qPCRs were performed in 96-well plates (VWR) with 4 μL of cDNA in 10-μL reactions prepared in duplicate using TB Green Premix Ex Taq II (RR820, Takara) on a Bio-Rad CFX-96 System. The qPCR conditions were 95°C for 7 minutes, 40 cycles of 95°C for 30 seconds, 59.5°C for 45 seconds, and 72°C for 30 seconds, followed by incubation at 95°C for 1 minute. To analyze transcription in mouse thymocytes, qPCR was performed in 96-well plates using 1 μL of cDNA and 0.24 μL of 50 X ROX in 12 μL reactions in duplicate using TB Green Premix Ex Taq II (RR820, Takara) on a StepOnePlus qPCR machine (Applied Biosystems). The qPCR conditions were 40 cycles of 95°C for 30 seconds and 59.5°C for 30 seconds, followed by incubation at 95°C for 1 minute. Melting curve analyses were performed with 55°C -90°C ramping in 0.5°C steps and 5-second increments to confirm a single amplicon for each sample and primer pair analyzed. The expression of individual genes was calculated using the ΔCt method and normalized to *Actb* transcription. All RT–qPCR experiments were performed with at least three biological replicates. The primers for *Actb*, *ACTB*, Cγ and Cδ transcripts have been previously described (14). The primers were obtained from Metabion and Integrated DNA Technologies, and their sequences are listed in **Table S1**. Primer sequences for eRNA detection are shown in **Table S1** and **Figure S2**.

### Analyses of assays for transposase-accessible chromatin using sequencing (ATAC-seq), chromatin immunoprecipitation using sequencing (ChIP-seq), and transcriptome (RNA-seq) databases

Guidelines for the design of primers for detection of eRNAs based on factor binding detection by ChIP-seq were previously described (55). To design the primers to detect eRNAs, we focused our search on the 250-500 bp sequences flanking the 324-bp mouse Eδ fragment, based on its homology with the equivalent human Eδ fragment, and the 227-bp mouse Eγ4 fragment, where functional transcription factors are known to bind (44, 56) (**Figures S2**, **S3**). To confirm that the designed primers are specific for detecting Eδ and Eγ4 transcripts, we analyzed chromatin profiles, transcript annotation, candidate *cis*-regulatory elements (cCREs), factor binding by ChIP-seq and RNA-seq in a 2.6-kb Eδ region and a 2.8-kb Eγ4 region using available databases (**Figures S2**, **S4-S6**). ATAC-seq data in DN2b and DN3 thymocytes and γδ T lymphocytes were retrieved from the Immunological Genome Project databrowsers (www.immgen.org) (57). Transcript annotation from GENCODE and the National Center for Biotechnology Information, cCREs from the ENCODE Registry, and transcription factor ChIP-seq information from ReMap Atlas of Regulatory Regions were retrieved using the UCSC Genome Browser. The ENCODE Registry of cCREs includes DNAseI hypersensitive sites across ENCODE samples that are supported by eH3K4me3, H3K27ac or CTCF binding by ChIP-seq. RNA-seq and H3K27ac ChIP-seq analyses in DN thymocytes were obtained from data series GSE80272 (58) and analyzed using Integrative Genomic Viewer (https://igv.org) (**Figure S6**).

### Electrophoretic mobility shift assays 

For use in EMSAs, SCID.adh cell extracts were obtained from 10^7^ unstimulated and mouse recombinant IL-7-stimulated cells for 30 minutes at 37°C. After washing with Hank´s balanced salt solution (Cultek), cells were resuspended in 200 mM NaCl, 50 mM Tris-HCl (pH: 8.0), 0.75 mM spermidine, 0.15 mM spermine, 0.1 mM EDTA, 0.1 mM Na_3_VO_4_, 1 mM DTT, 0.5 mM PMSF, and 1X complete protease inhibitors (Roche, Merck), lysed by adding Nonidet-40 to a 10% solution to a final concentration of 0.4% and incubated for 30 minutes on ice. Lysates were clarified by centrifugation at 12,000 × g for 10 minutes at 4°C, and glycerol was added to a final proportion of 25%. The protein concentration was determined by the Bradford assay (Bio-Rad). A total of 60,000 cpm of ^32^P-labeled double-stranded oligonucleotide was incubated with 12 μg of cell extract in a 25-μL volume containing 10 mM Tris-HCl (pH 7.5), 50 mM NaCl, 1 mM EDTA, 2% glycerol, 1 μg of poly(dI-dC), and 1 μg of bovine serum albumin for 20 minutes on ice. One microgram of anti-STAT5 antibody (Santa Cruz Biotechnology, sc-835), which recognizes STAT5a and STAT5b, was added and incubated for 30 minutes at room temperature to supershift the specific complex. The binding sites are listed in **Table S1**. Native polyacrylamide (4.5%) containing bis-acrylamide/acrylamide (1:19) containing 0.25X Tris-borate-EDTA previously run at 200 V for 1 hour was used to separate the DNA and DNA/protein complexes. The gels were fixed with 30% methanol and 10% acetic acid for 30 minutes and then dried and exposed to film. The primers of the tested binding sites were obtained from Metabion, and the sequences are listed in **Table S1**.

### Quantitative chromatin immunoprecipitation 

qChIP experiments were performed with chromatin from 10^7^ cells incubated with 5 μg of anti-STAT5 (Santa Cruz Biotechnology, sc-235), trimethylated lysine 4 of histone H3 (H3K4me3) (ab8580, Abcam), acetylated lysine 27 of histone H3 (H3K27ac) (ab4779, Abcam), or control (clone 1-1, Millipore, Merck or ab46540, Abcam) antibodies as previously described (14). The primers used for Eγ, Eδ, the *Tcra* enhancer (Eα), and *Oct2* exon in the qChIP have been previously described (14, 59). The primers were obtained from Metabion, and the sequences are listed in **Table S1**.

### Luciferase assays

Reporter plasmids containing the firefly luciferase reporter gene driven only by a human TRDV1 promoter (Vδ1p) alone and a human 370-bp Eδ fragment driven by Vδ1p were constructed based on the pXPG plasmid as previously described (60). Reporter plasmids containing the firefly luciferase reporter gene driven only by a minimal murine *Fos* promoter (cfosp) and by cfosp with murine 410-bp Eγ1 were constructed based on the pGL4.10 plasmid (Promega) as previously described (14, 44). To introduce a point mutation in the STAT5-binding site present in the δE6/7 region (δE6/7) of the Eδ370-Vδ1p-luciferase plasmid, a Q5 site-directed mutagenesis kit (E0554, New England Biolabs) was used with HPLC purified primers designed by the NEBaseChanger program. The sequences of the primers used are listed in **Table S1**. The mutation was confirmed by DraI digestion and sequencing. For luciferase assays, 5x10^6^ Jurkat-GFP or Jurkat-IL7Rα cells were transfected by electroporation with 5 μg of the firefly luciferase reporter plasmid and 10 ng of the pRL-TK (Promega) Renilla luciferase reporter plasmid. Both electroporation and measurements of firefly and Renilla luciferase activities were performed as previously described (14).

### Statistical analysis

Statistical analysis was performed with Prism 5.0 software (GraphPad). At least three independent experiments were performed in all cases. The number of independent experiments analyzed (n) is indicated in the figure legends. Nonparametric unpaired Student´s t tests with the Welch correction were performed, and significant differences between the indicated values are indicated by asterisks as follows: *p <*0.05 (*), *p* < 0.005 (**), and *p*<0.0005 (***). The absence of an asterisk indicates that the change relative to the control was not statistically significant.

## Results

### IL-7R signaling activates *Tcrd* germline transcription in DN3a thymocytes

**Figure 1:**
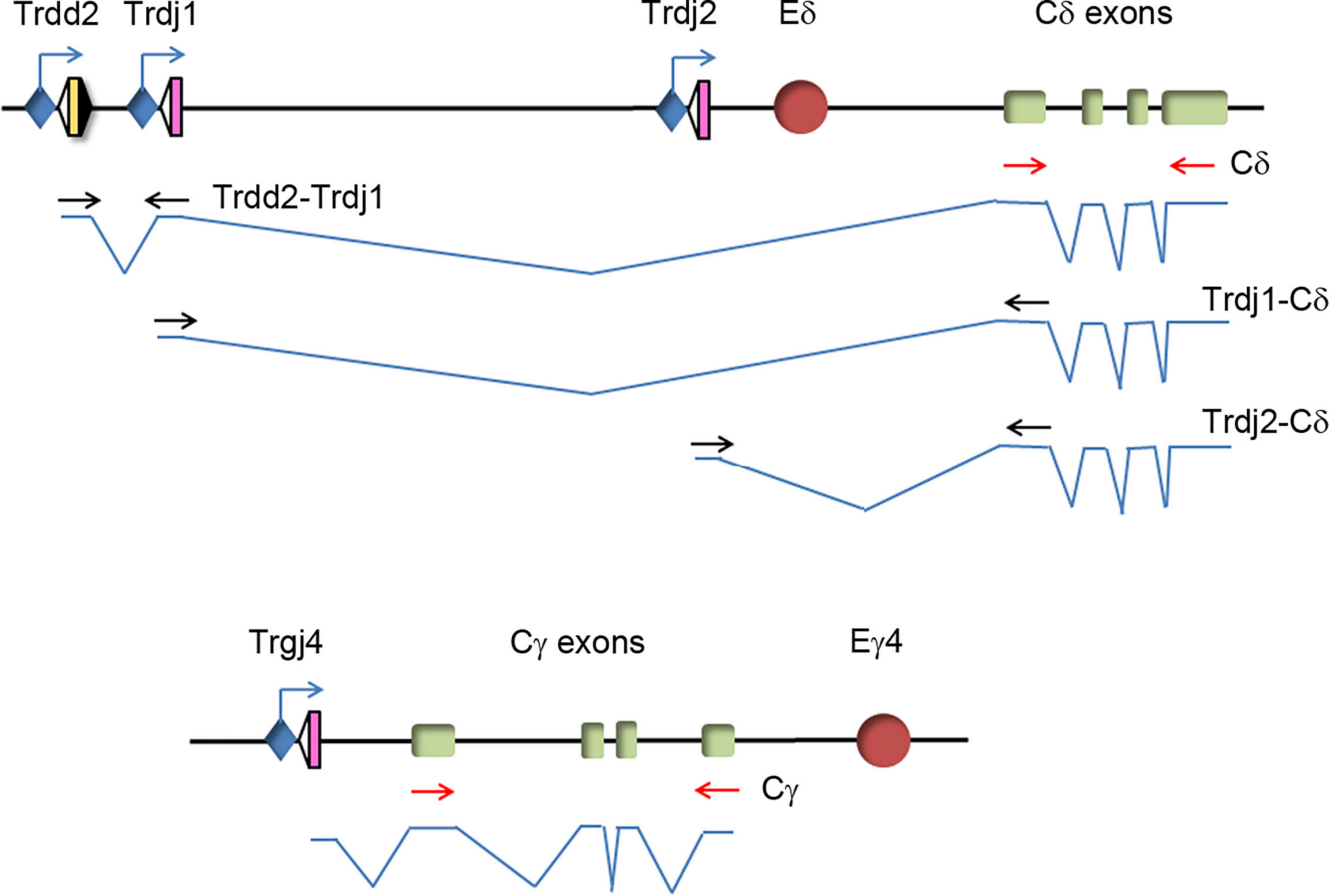
Structure of *Tcrd* and *Tcrg* germline transcripts. Transcription and splicing are indicated by blue lines. The position of primers used to detect specific germline transcripts is indicated: primers used to detect Cδ and Cγ transcripts are represented in red, and primers to detect Trdd2-Trdj1, Trdj1-Cδ and Trdj2-Cδ transcripts are represented in black.

**Figure 2:**
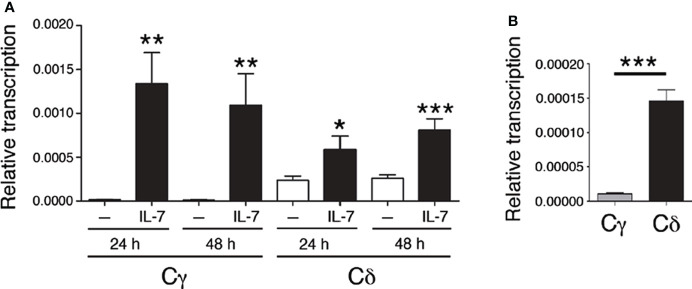
IL-7 activates *Tcrg* and *Tcrd* germline transcription. **(A)** Analysis of Cγ and Cδ transcription in untreated (-, white bars) and IL-7-treated SCID.adh cells (IL-7, black bars) after 24 or 48 hours, as indicated, as determined by RT–qPCR. **(B)** Transcriptional analysis of Cγ and Cδ in untreated SCID.adh cells cultured for 24 hours, as determined by RT–qPCR. The results were normalized to those of *Actb* and represent the mean ± standard error of the mean (SEM) of duplicate RT–qPCRs based on 8 independent experiments. Nonparametric unpaired Student´s t tests with the Welch correction were performed, and *p* values are represented by asterisks as follows: *p*<0.05 (*), *p*<0.005 (**), and *p*<0.0005 (***). The significance of the difference between values obtained with untreated and IL-7-treated cells is shown.

*Tcrd* is flanked by *Tcra* Vα and Jα gene segments and comprises Vδ gene segments interspaced with Vα segments within an ~1 Mb region, followed by a 33.7-kb region that contains two Dδ (Trdd1 and Trdd2) gene segments, two Jδ (Trdj1 and Trdj2) gene segments, Eδ, the *Tcrd* C region (Cδ), and the inverted Trdv5 gene segment in murine chromosome 14 (9) (**Figure S1**). *Tcrg* spans 0.2 Mb and comprises three functional Vγ-Jγ-*Tcrg* C region (Cγ1, Cγ2 or Cγ4)-Eγ clusters in murine chromosome 13 (9) (**Figure S1**). Expression of the TCRγ and TCRδ chains results from the activation of enhancer-dependent germline transcription of their respective unrearranged genes, which induces long-range chromatin changes that trigger VγJγ and VδDδJδ recombination in DN2b and DN3a thymocytes (**Figure S1**). These noncoding transcripts are initiated by promoters associated with the Jγ, Dδ, and Jδ gene segments that are ultimately spliced into their respective constant regions (40, 61) (**Figure 1**). The levels of germline transcription measured at these constant regions represent the sum of all the transcripts that are initiated in the D and/or J gene segments in their respective gene or gene cluster. To evaluate the potential role played by IL-7R signaling in the activation of *Tcrd* germline transcription, cells of the appropriate developmental stage that are deficient in V(D)J recombination must be used. We analyzed the levels of Cδ transcripts in untreated and IL-7-treated SCID.adh cells and compared them with the well-known regulation of *Tcrg* germline transcription by measuring IL-7-dependent activation of Cγ transcripts (40, 41) (**Figure 2A**). These cells, which were derived from mice carrying an inactivating spontaneous point mutation in the catalytic subunit of DNA-PK, exhibit a DN3a-like phenotype derived from their complete defect in V(D)J recombination (50, 62). Because their TCR genes are in a germline unrearranged configuration, these cells constitute an excellent model with which to study IL-7R-dependent *Tcrg* transcription, as well as pre-TCR-induced silencing of *Tcrg* and *Tcrd* and activation of *Tcra* (14, 16, 44, 45, 60, 63). Due to a deletion at the 5´-end of the *Tcrg* locus in these cells, germline transcription of Cγ4 (Cγ) was analyzed as representative of the three Vγ-Jγ-Cγ clusters because they share the same regulation (16) (**Figure 1**). Although basal Cδ transcription was found to be higher than Cγ transcription in these cells, IL-7 treatment clearly induced both Cγ and Cδ transcription (**Figures 2A, B**).

### IL-7R-dependent activation of *Tcrg* and *Tcrd* germline transcription is regulated by Notch signaling

**Figure 3:**
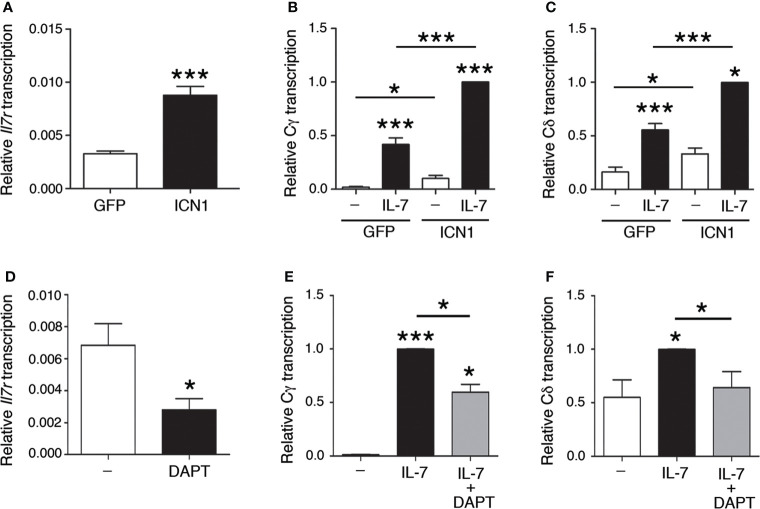
Notch-dependent regulation of IL-7R-dependent *Tcrg* and *Tcrd* germline transcription. RT–qPCR analysis of **(A)** *Il7ra*, **(B)** Cγ, and **(C)** Cδ transcription (n=8, n=3, and n=3, respectively) in SCID.adh cells transduced with GFP (GFP) or ICN1 + GFP (ICN1) retroviruses and incubated in the absence **(-)** or presence of IL-7 (IL-7), as indicated. RT–qPCR analysis of **(D)** *Il7ra*, **(E)** Cγ, and **(F)** Cδ transcription (n=3) in untreated or DAPT-treated SCID.adh cells incubated in the absence **(-)** or presence (IL-7) of IL-7, as indicated. The results were normalized to those of *Actb* and represent the mean ± SEM of duplicate RT–qPCRs in the indicated number (n) of independent experiments. Nonparametric unpaired Student´s t tests with the Welch correction were performed, and *p* values are represented by asterisks as follows: *p*<0.05 (*) and *p*<0.0005 (***). Significant differences between the obtained values in cells untreated or treated with IL-7, transduced with GFP or ICN1 + GFP retroviruses or untreated and DAPT-treated cells as indicated are shown.

Regulation of enhancer-dependent *Tcrg* and *Tcrd* germline transcription is regulated by Notch signaling (14). Because IL-7Rα is a target of Notch signaling (14, 29–31), we evaluated the effect of gain and loss of NOTCH1 signaling on *Il7ra-* and IL-7-dependent Cγ and Cδ transcription (**Figure 3**). As expected (14), transduction of SCID.adh cells with intracellular NOTCH1 domain (ICN1)-expressing retroviruses induced *Il7ra* transcription (**Figure 3A**). Accordingly, IL-7-dependent activation of Cγ and Cδ transcription was induced in SCID.adh cells that had been transduced with ICN1 + GFP-expressing retroviruses, and the transcription levels were compared with those of cells that had been transduced with retroviruses that expressed only GFP (**Figures 3B, C**). In contrast, cell treatment with the γ-secretase inhibitor DAPT, which inhibits proteolytic cleavage and thus prevents the release of endogenous ICN1, inhibited *Il7ra* transcription (**Figure 3D**); therefore, a decrease in IL-7-dependent activation of Cγ and Cδ transcription was detected (**Figures 3E, F**). These results indicate that the Notch-dependent effect on *Il7ra* transcription causes increased activation of IL-7-dependent *Tcrd* and *Tcrg* germline transcription. These results confirm that the transcriptional regulatory axis formed by the NOTCH1 and IL-7R pathways was evident in SCID.adh cells and involved in the regulation of *Tcrg* and *Tcrd* transcription in DN3a thymocytes. Therefore, the mechanism for the regulation of *Tcrg* and *Tcrd* germline transcription by this regulatory axis is based on the regulation of IL-7R expression by Notch signaling, which results in increased responsiveness of the unrearranged *Tcrg* and *Tcrd* genes to IL-7.

### IL-7R signaling is essential for *Tcrd* germline transcription *in vivo*

**Figure 4:**
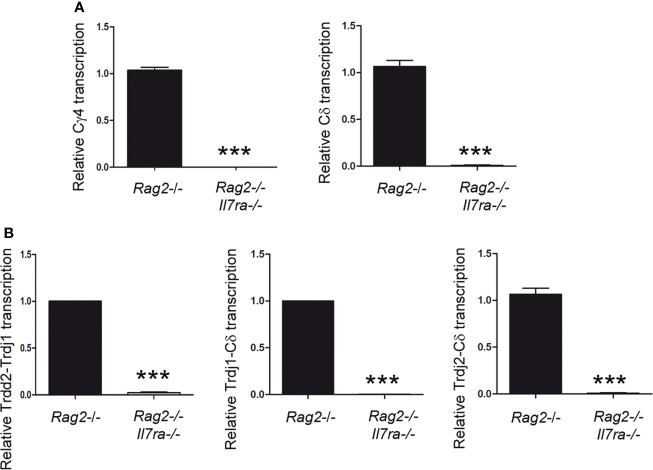
IL-7R signaling is essential for *Tcrd* germline transcription *in vivo*. Analysis of **(A)** Cγ and Cδ and **(B)** Trdd2-Trdj1, Trdj1-Cδ and Trdj2-Cδ transcripts in *Rag2* ^-/-^ and *Rag2* ^-/-^ *Il7ra* ^-/-^ thymocytes by RT–qPCR. The results were normalized to those of *Actb* and represent the mean ± SEM of duplicate RT–qPCRs based on 3 independent experiments. Nonparametric unpaired Student´s t tests with the Welch correction were performed, and *p*<0.0005 values based on values obtained with *Rag2* ^-/-^ and *Rag2^-/-^Il7ra^-/-^* thymocytes are represented by asterisks as *** (*p*<0.0005).

To study TCR germline transcription, thymocytes of the appropriate developmental stage (such as DN3a in the case of *Tcrg* and *Tcrd*) that are deficient in V(D)J recombination must be analyzed. *Rag2* ^-/-^ mice show deficient V(D)J recombination; therefore, thymocyte development is blocked at the DN3a stage in these mice (21, 49, 64). In fact, these animals constitute a pure source of DN3a thymocytes, 99.0 ± 0.8% of total thymocytes (64). The *Tcrg* and *Tcrd* in an unrearranged configuration in these mice allowed us to analyze germline transcription in DN3a thymocytes. To clearly determine the role played by IL-7R signaling *in vivo*, we compared Cγ and Cδ germline transcription in *Rag2* ^-/-^ and *Rag2* ^-/-^ *Il7ra* ^-/-^ DN3a thymocytes by performing RT–qPCR (**Figure 4A**). Because *Rag2* ^-/-^ thymocyte blockade occurs earlier during development and predominates over *Il7ra* deficiency (28, 49), both *Rag2* ^-/-^ and *Rag2* ^-/-^ *Il7ra* ^-/-^ mice have an equivalent block at the DN3a stage. As expected (40), Cγ transcription was abrogated in *Rag2* ^-/-^ *Il7ra* ^-/-^ DN3a thymocytes. Our analyses of Cδ transcription indicated that *Tcrd* germline transcription was also strongly dependent on IL-7R signaling (**Figure 4A**). Cδ transcripts constitute the sum of *Tcrd* germline transcripts initiated at the Trdd2, Trdj1, and Trdj2 promoters (**Figure 1**). We also analyzed specific transcripts initiated at each of these promoters. The transcripts initiated at the Trdj1 and Trdj2 promoters are spliced to the first exon of Cδ, while those initiated at the Trdd2 promoter are first spliced to the Trdj1 gene segment before splicing to the Cδ first exon (**Figure 1**). The Trdd2-Trdj1, Trdj1-Cδ and Trdj2-Cδ transcripts were clearly detected in *Rag2* ^-/-^ thymocytes (**Figure 4B**). According to the strong inhibition of Cδ transcription, the aforementioned transcripts were profoundly inhibited in *Rag2* ^-/-^ *Il7ra* ^-/-^ thymocytes (**Figure 4B**). These results indicate that, similar to *Tcrg* germline transcription, *Tcrd* germline transcription depends on IL-7R signaling in DN3a thymocytes.

### STAT5 binds to Eδ

**Figure 5:**
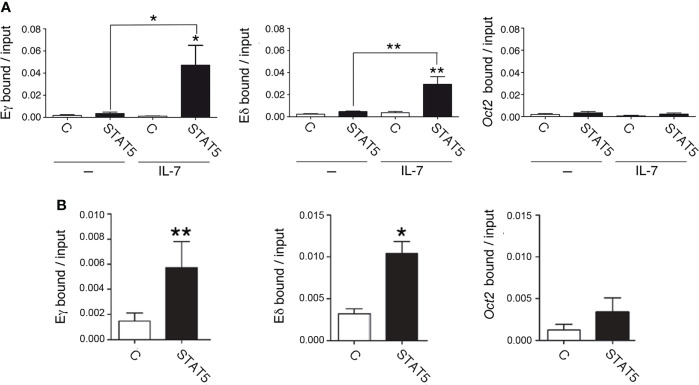
IL-7R signaling induces STAT5 recruitment to Eγ and Eδ in DN3a thymocytes. **(A)** Binding of STAT5 to Eδ, Eγ4 and *Oct2* sequences in untreated (-) and IL-7-treated (IL-7) SCID.adh cells determined after 30 minutes by qChIP (n=8). **(B)** Binding of STAT5 to Eδ, Eγ4 and *Oct2* sequences in *Rag2* ^-/-^ thymocytes as determined by qChIP (n=4). The data represent the mean ± SEM of duplicate results obtained from n independent qChIP experiments. Nonparametric unpaired Student´s t tests with the Welch correction were performed as indicated, and *p* values are represented by asterisks as follows: *p*<0.05 (*) and *p*<0.005 (**). Significance between the values obtained using an anti-STAT5 antibody (STAT5) and control antibody (C), as indicated, is shown.

IL-7R signaling results in rapid phosphorylation of STAT5, which is translocated from the cytoplasm to the nucleus to activate its target genes. Accordingly, IL-7R signaling activates Cγ transcription through the recruitment of STAT5 to Eγ (44). We compared STAT5 binding to Eγ4 and Eδ by qChIP in unstimulated and IL-7-stimulated SCID.adh cells after a 30-minute treatment (**Figure 5A**). We found comparable STAT5 recruitment to both Eγ4 and Eδ upon IL-7 treatment. To confirm the recruitment of STAT5 in primary DN3a cells, we evaluated its binding in *Rag2* ^-/-^ thymocytes (**Figure 5B**). IL-7 treatment was not necessary to detect STAT5 binding to these enhancers in ex-vivo *Rag2* ^-/-^ thymocytes, most likely because these cells were already stimulated *in vivo*. We found similar STAT5 binding to both enhancers, confirming the results obtained with SCID.adh cells. As a negative control in our qChIP experiments, STAT5 binding to an *Oct2* exon sequence was also analyzed (**Figures 5A, B**).

### IL-7R signaling activates Eδ function through STAT5 binding to the δE6/7 site

**Figure 6:**
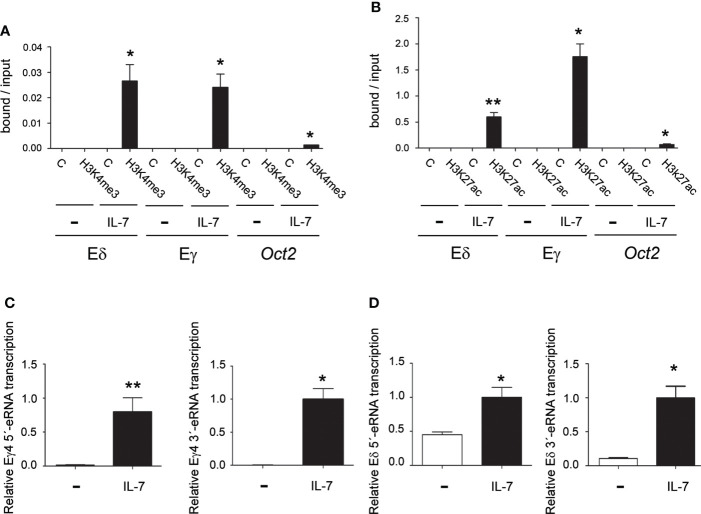
IL-7R signaling activates Eγ and Eδ epigenetic marks and eRNA transcription. Analyses of H3K4me3 **(A)** and H3K27ac **(B)** histone marks in Eγ4, Eδ and *Oct2* sequences in untreated (-) and IL-7-treated (IL-7) SCID.adh cells as determined after 24 hours by qChIP. The data represent the mean ± SEM of duplicate results obtained from 4 independent qChIP experiments. Nonparametric unpaired Student´s t tests with the Welch correction were performed as indicated, and the significance of differences between the values obtained using anti-H3K4me3 or anti-H3K27ac and control antibodies (C) are shown. Analyses of Eγ4 **(C)** and Eδ **(D)** eRNA transcription in untreated (-) and IL-7-treated (IL-7) SCID.adh cells after 24 hours by RT–qPCR. The results were normalized to those of *Actb* and represent the mean ± SEM of duplicate RT–qPCRs from 3 independent experiments. Nonparametric unpaired Student´s t tests with the Welch correction were performed, and the significance of differences between the values obtained with untreated and IL-7-treated cells is shown. *p* values are represented by asterisks as follows: *p*<0.05 (*) and *p*<0.005 (**).

eRNAs together with epigenetic activation marks on histone H3, such as trimethylation of lysine 4 (H3K4me3) and acetylation of lysine 27 (H3K27ac) are predictors of enhancer activity (65–68). To evaluate whether IL-7 treatment can directly activate Eδ and Eγ activity, we analyzed the effect of IL-7R signaling on H3K4me3 and H3K27ac on Eδ and Eγ4 in unstimulated and IL-7-treated SCID.adh cells (**Figures 6A, B**). Consistent with the presence of these chromatin marks on active enhancers (67, 68), we found that H3K4me3 and H3K27ac modification was strongly induced at both enhancers, but not at a negative control sequence, after IL-7 stimulation of SCID.adh cells. Detection of eRNAs is the most reliable indicator of enhancer activity (65, 66). These noncoding transcripts are unidirectional or bidirectional and have low abundance due to their instability. Enhancer activation correlated with IL-7-dependent induction of bidirectional Eδ and Eγ4 eRNAs in SCID.adh cells (**Figures 6C, D**). To examine the presence of other *cis*-regulatory regions in the surrounding enhancer regions, we analyzed chromatin accessibility in DN2b and DN3 thymocytes, and γδ T lymphocytes by ATAC-seq using the Immunological Genome Project databrowsers (www.immgen.org) (57), as well as the presence of other enhancers in the vicinity according to the ENCODE Registry of cCREs (**Figures S2**, **S4**, **S5**). Although these analyses indicate the presence of other *cis*-regulatory elements located in the vicinity of Eδ and Eγ4 within a region of less than 2 kb, these enhancers constitute the sequences with the highest density of transcription factor binding by ChIP-seq according to ReMap Atlas of regulatory regions (**Figures S4**, **S5**). Interestingly, p300 and STAT5 are specifically recruited to these enhancers (**Figures S4**, **S5**). Taken together, these data clearly demonstrate that Eγ and Eδ are both activated by IL-7R signaling.

**Figure 7:**
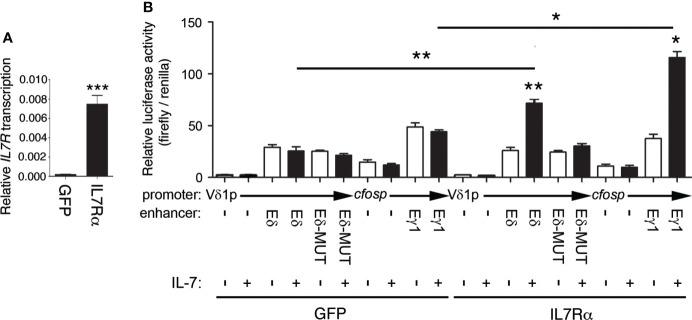
Eδ is activated by IL-7R signaling. **(A)** Transcriptional analysis of IL7R in GFP- (GFP) and IL7Rα-GFP- (IL7Rα)-expressing Jurkat cells. The results were normalized to those of *ACTB* and represent the mean ± SEM of duplicate RT–qPCRs based on 3 independent experiments. The significance of differences between the values obtained with GFP- and IL7Rα-GFP-expressing Jurkat cells is shown. **(B)** Transcriptional analysis of Eδ-, Eδ-MUT- and Eγ1-dependent luciferase constructs transfected into GFP- (GFP)- or IL7Rα-GFP-(IL7Rα)-expressing Jurkat cells that were untreated (-) or treated with IL-7 (+) for 48 hours. The data represent the mean ± SEM of duplicate results obtained from 6 independent firefly/Renilla luciferase assays. The significance of differences between the values obtained from untreated and IL-7-treated cells, as indicated, is shown. Nonparametric unpaired Student´s t tests with the Welch correction were performed, and *p* values are represented by asterisks as follows: *p*<0.05 (*), *p*<0.005 (**), *p*<0.0005 (***).

To directly evaluate the role of IL-7 on Eδ function, we analyzed its effect on enhancer activity using luciferase reporter constructs in transiently transfected Jurkat cells (**Figure 7**). These cells constitute a well-established model for studying TCR enhancer activity upon cell stimulation (14, 60). Because these cells express very low levels of IL7Rα, we used two Jurkat clones that had been previously obtained through retroviral transduction and that expressed GFP or IL7Rα + GFP (53). As shown in **Figure 7A**, the GFP-expressing cells exhibited very low levels of *IL7RA* expression compared with the cells transduced with GFP + IL7Rα-expressing retroviruses. As expected, Eγ activity was highly activated by IL-7 treatment in the IL7Rα + GFP-expressing cells but not in the control GFP-expressing cells (**Figure 7B**). Similarly, we found that Eδ activity was activated by IL-7 only in the IL7Rα + GFP-expressing cells and not in the control GFP-expressing cells (**Figure 7B**). The observed effects were clearly mediated by the respective enhancer because the luciferase activity of the constructs with no enhancer in either clone was unaffected by IL-7 treatment. Of the two conserved putative STAT5 sites found by comparing murine and human Eδ sequences (**Figure S3**), we validated by EMSA the STAT5-binding site that was located between δE6 and δE7, the δE6/7 site (**Figures S7A, B**). STAT5 binding to this site is consistent with recruitment data for this factor to Eδ by ChIP-seq in immune cells and tissues based on ReMap Atlas of Regulatory Regions (**Figure S4**). Introduction of a mutation that abolished STAT5 binding to the human δE6/7 site (**Figure S7C**) abrogated enhancer activation by IL-7 treatment in IL7Rα-expressing transfected Jurkat cells (**Figure 7B**). Together, our results demonstrate that, in addition to the regulation of *Tcrg* germline transcription and Eγ function, IL-7R signaling is crucial for Eδ-dependent *Tcrd* germline transcription.

## Discussion

Eγ and Eδ are regulated in parallel during β-selection, activating germline transcription and VγJγ and VδDδJδ recombination in DN2b and DN3a thymocytes and gene silencing in DP thymocytes (15, 69). Pre-TCR signaling causes dissociation of Eγ- and Eδ-bound factors in DP thymocytes (14–16). MYB and RUNX1 dissociate from Eγ and Eδ during β-selection as a result of the pre-TCR-dependent downregulation of *Notch1* transcription (14, 32), whereas STAT5 dissociates from Eγ as a result of terminated *Il7ra* transcription (16). In this study, we demonstrate that Eδ function depends on IL-7R-dependent STAT5 recruitment, similar to the mechanism of Eγ function induction, demonstrating a parallel regulatory mechanism of these enhancer functions in controlling *Tcrg* and *Tcrd* germline transcription. Hence, the activity of Eγ and Eδ depends on RUNX1, MYB, and STAT5 recruitment in DN3a thymocytes, whereas these three aforementioned factors dissociate from Eγ and Eδ in DP thymocytes as a consequence of termination of Notch and IL-7R signaling, revealing the molecular mechanism by which *Tcrg* and *Tcrd* transcription is regulated in parallel during thymocyte development.

To study the role of the combined effect of IL-7 and Notch signaling, we analyzed the effect of IL-7 on ICN1-transduced SCID.adh cells. These cells produce full-length and truncated *Notch1* transcripts, which derive from an intragenic deletion of approximately 38 kb and consist of exon 1 joined to an 81-kb noncontiguous intron 1 sequence that it is spliced to a site 12 bp 3´ of the exon 28 splice acceptor site (70). The resulting polypeptide can insert into the cell membrane due to its hydrophobic N-terminus, driving ICN1 expression to generate ligand-independent signals in a DAPT-sensitive fashion. Previous studies have demonstrated that SCID.adh cells constitutively express some levels of ICN1 and respond to DAPT by downregulating ICN1 expression as well as Notch-dependent genes, such as *Cd25*, *Hes1*, *Il7ra*, *Runx1*, *Tcrd* and *Tcrg* (14, 70, 71). In addition, these cells respond to IL-7 signaling and have been previously used to analyze its role in regulating *Tcrg* germline transcription (14, 44, 45). Therefore, SCID.adh cells constitute an excellent model to study the combined effect of Notch and IL-7 in the regulation of *Tcrg* and *Tcrd* germline transcription. Consistent with the induction of *Il7ra* transcription by Notch, our results revealed that IL-7-dependent activation of *Tcrd* and *Tcrg* germline transcription was further activated by ICN1 and inhibited by DAPT in these cells. These results strongly suggest that the Notch-dependent effect on *Il7ra* transcription is responsible for the IL-7-dependent *Tcrd* and *Tcrg* germline transcription observed upon ICN1 overexpression and DAPT treatment in SCID.adh cells.

To demonstrate the essential role for IL-7 signaling in activating Eδ and Eγ4 in SCID.adh cells, we analyzed H3K4me3 and H3K27ac together with the induction of eRNAs as predictors of enhancer activity (65–68). In fact, enhancer transcription is considered the best indicator of enhancer activity (65, 66). The detection of IL-7-induced Eγ and Eδ transcripts indicates that this treatment induces an opening in the chromatin structure at the enhancer regions. Although other open regions that could function as *cis*- regulatory elements are present in the vicinity of Eδ and Eγ4, as indicated by ATAC-seq and the ENCODE Registry of cCREs, these enhancers concentrate the highest binding density of transcription factors, including the specific binding of p300 and STAT5 (**Figures S2**, **S4**, **S5**). This high density of transcription factors that bind to Eδ and Eγ4 is consistent with the absence of H3K27ac at the core site of these enhancers, with this histone mark detected in the flanking regions of these enhancers (**Figures S4-S6**). These analyses confirm that Eδ and Eγ4 are the main regulatory elements present in the regions analyzed. Because STAT5 specifically binds to Eδ and Eγ4 and not to other nearby enhancers as analyzed by ChIP-seq based on ReMap Atlas of Regulatory Regions, our data indicating that the measured transcripts are IL-7 responsive strongly support that they constitute true Eδ and Eγ4 eRNAs. Although the distal enhancer EO581865/enhD, located adjacent to Eδ at a distance of approximatelly 100 pb, exhibits some levels of STAT5 binding based on ReMap data, ATAC-seq experiments indicate that the EO581865/endD chromatin is not accessible in DN2b and DN3 thymocytes, indicating that Eδ is the relevant enhancer at the *Tcrd* locus during thymocyte development (**Figure S4**). The low levels of transcripts detected in Eδ and Eγ4 surrounding regions by RNA-seq in DN thymocytes are consistent with the expected low abundance of eRNAs (**Figure S6**). Although the role of eRNAs remains unresolved, they are thought to be relevant to maintaining an open chromatin state that is readily accessible for transcription factors, stabilizing enhancer-promoter looping interactions, promoting the loading of RNA-polymerase 2 to the promoter, and/or releasing a paused promoter to an elongating stage (72–74). Our experiments do not address the potential roles of these eRNAs on *Tcrd* and *Tcrg* transcription, but these transcripts likely contribute to maintaining the opening of enhancer chromatin to facilitate access to transcription factors and cofactors in the activation of their specific promoters.

Previous experiments with *Il7ra* ^-/-^ mice demonstrated a strong dependence on IL-7R signaling in the regulation of *Tcrg* germline transcription and VγJγ recombination and little apparent effect on *Tcrd* recombination (38–42, 48). Although a partial inhibitory effect on VδDδJδ might be overlooked in these experiments (38), these results differ from our results, with dramatically reduced Trdd2, Trdj1 and Trdj2 germline transcription observed in *Rag2* ^-/-^ *Il7ra* ^-/-^ DN3a thymocytes and IL-7R-dependent regulation of Eδ. Consistent with the important role played by Eδ in promoting chromatin accessibility and activating Trdd2, Trdj1 and Trdj2 germline transcription in a discrete chromatin loop (75), previous experiments with Eδ^-/-^ mice demonstrated that this enhancer is important for *Tcrd* germline transcription and VδDδJδ rearrangements in DN3a thymocytes and the generation of γδ T lymphocytes (23). Because *Tcrd* germline transcription primarily depends on Eδ function (23), our results demonstrate that IL-7R signaling plays a crucial role in the control of Eδ-dependent *Tcrd* germline transcription in DN3a cells. Therefore, the VδDδJδ rearrangements detected in *Il7ra* ^-/-^ mice are most likely the consequence of a very low level of *Tcrd* germline transcription in *Rag2* ^-/-^ *Il7ra* ^-/-^ thymocytes; this low level of transcription may open the locus chromatin to permit accessibility of the recombinase machinery. Although profoundly reduced compared to the levels in the control mice, 10-12% thymic and 6-10% splenic γδ T lymphocytes were detected in the Eδ^-/-^ mice; the presence of some γδ T cells in Eδ^-/-^ mice suggested the implication of additional elements in activating VδDδJδ recombination. Our data indicate that nearly all *Tcrd* germline transcripts were abrogated in *Rag2* ^-/-^ *Il7ra* ^-/-^ thymocytes compared to *Rag2* ^-/-^ thymocytes, including those initiated by the Trdd2 promoter (**Figure 4**), which had been previously proposed to be a possible candidate for promoting *Tcrd* germline transcription and VδDδJδ recombination in Eδ^-/-^ mice (61). The detection of only residual *Tcrd* transcripts in our analysis of *Rag2* ^-/-^ *Il7ra* ^-/-^ thymocytes does not support a suggestion of additional IL-7R-independent regulatory elements in the activation of VδDδJδ recombination. Since our experiments were focused on the regulation of *Tcrd* and *Tcrg* germline transcription that occur prior to VδDδJδ and VγJγ rearrangements and thus TCRγδ expression, these data do not directly address the issue of αβ vs. γδ T-cell commitment, which is accepted to be regulated by differential signaling strength between TCRγδ and pre-TCR expressed on the same T-cell precursors (11–13).

In contrast to its important function in *Tcrd* germline transcription and VδDδJδ recombination in thymocytes, Eδ is not required for the transcription of a rearranged *Tcrd* gene in mature γδ T lymphocytes; in fact, Eα is the regulatory element critical for this transcriptional function (23, 76). IL-7R signaling does not activate *Tcra* germline transcription or induce STAT5 binding to Eα in SCID.adh cells (**Figures S8A, B**). In contrast, IL-7R signaling has been previously shown to be involved in preventing premature VαJα recombination in DN4 thymocytes (28). Therefore, IL-7R signaling is probably not required for rearranged *Tcrd* transcription in mature γδ T cells. Because Eδ is critical for the premature VαJα rearrangements that have been detected in Eα^-/-^ mice that result in the detection of Vα2^+^ T lymphocytes in these mice (76–78), NOTCH1 and IL-7R signaling likely regulate the induction of Eδ-dependent VαJα rearrangements in DN3a thymocytes.

Comparisons between synthetic and natural enhancers have revealed that enhancer activity is best explained by occupancy of specific binding sites regardless of the binding site position (79). Hence, the combination of multiple transcription factor-binding sites and not their organization underlies the specificity of eukaryotic gene expression regulation (80). In addition, temporal expression of specific transcription factors clearly regulates T-lineage identity and development (24). In this regard, the combination of the essential binding sites for RUNX1, MYB and STAT5 is conserved between Eγ and Eδ in both mice and humans; however, these sites are positioned differently from STAT5-MYB-RUNX1 sites in Eγ and RUNX1-MYB-STAT5 sites in Eδ (**Figure S7**). Therefore, the recruitment of these factors to differently organized binding sites within these enhancers could create efficient regulatory structures that are critical for high *Tcrg* and *Tcrd* gene expression in DN2b and DN3a thymocytes and gene silencing in DP thymocytes and αβ T lymphocytes. We have not directly addressed the effect of IL-7 on the recruitment of RUNX1 and MYB to Eδ in SCID.adh cells; however previous studies have shown that IL-7 treatment does not inhibit the recruitment of RUNX1 and MYB to Eγ in these cells (44). The fact that RUNX1, MYB and STAT5 bind to Eγ in IL-7-treated SCID.adh cells (44) as well as to Eγ and Eδ in *Rag2* ^-/-^ thymocytes (14, 16) supports the hypothesis that these factors are simultaneously recruited to these enhancers in DN3a thymocytes. Furthermore, luciferase assays indicated that STAT5 binding synergistically augmented the activity of Eγ activity along with RUNX1 and MYB (44), and IL-7 treatment increased Eδ activity (**Figure 7**), which is absolutely dependent on the presence of intact RUNX1 and MYB binding sites (33–36). Together, these results strongly suggest that these factors are simultaneously recruited to Eδ and Eγ to regulate enhancer function in DN3a thymocytes.

The functional interconnection of the IL-7R and NOTCH1 signaling pathways is essential for normal T-cell development. When this intersection is defective, lymphopenia can be a result, whereas excessive signaling can lead to the development of T-cell acute lymphoblastic leukemia (81). In fact, constitutive activation of *NOTCH1* signaling is the most prominent oncogenic pathway during T-cell transformation in more than 60% of all human T-cell acute lymphoblastic leukemia cases, which are mainly caused by different activating mutations (82). Interestingly, in 70% of the latter, chromosomal translocations are evident during thymocyte development as a result of illegitimate TCR gene recombination, with those involving *TCRD* predominant in approximately 67% of cases (83, 84) and Eδ being an important element contributing to genomic instability (85). Our results revealing an important role played by IL-7R signaling in the regulation of Eδ-dependent *Tcrd* germline transcription in DN3a thymocytes might contribute to a better understanding of the causes of this disease.

## Data availability statement

The raw data supporting the conclusions of this article will be made available by the authors, without undue reservation.

## Ethics statement

The animal study was reviewed and approved by Ethical Committee of Consejo Superior de Investigaciones Científicas, Spain Ethical Commitee of Andalusian Government, Spain Animal Care and Use Commitee of Kyoto University, Japan. Written informed consent was obtained from the owners for the participation of their animals in this study.

## Author contributions

AR-C performed and analyzed the experiments. ST-I and KI provided the data shown in **Figure 4**. ÁC and AR-C performed the experiments shown in **Figure S8**. JL-R participated in experiments performed with the mice. CS participated in the interpretation of the results. CH-M designed the research, analyzed the results, made the figures and wrote the article. All authors contributed to the article and approved the submitted version.

## Funding

This work was funded by grants from the Spanish Ministry of Science and Innovation (BFU2016-79699P and PID2021-128720NB-100), Spanish Scientific Research Council (2019AEP202), and Andalusian Government (P20_01271) to CH-M; the Spanish Ministry of Science and Competitiveness (PID2020-118859GB-100) and Andalusian Government (P20_01269) to CS; and JSPS KANENHI (19K08999) to ST-I. This research was co-funded with European Union funds. AR-C, JL-R, and CH-M are part of CSIC´s Global Health Platform (PTI+ Salud Global) (SGL2103033).

## Acknowledgments

We thank Nuno L. Alves (Institute for Molecular and Cell Biology, Porto, Portugal) and René A. W. van Lier (Academic Medical Center, Amsterdam, The Netherlands) for the Jurkat-GFP and Jurkat-IL7Ra + GFP clones; José Zamorano (San Pedro de Alcántara Hospital, Cáceres, Spain) for his help in STAT5 EMSAs; David L. Wiest (Fox Chase Cancer Center, Philadelphia, PA, USA) for SCID.adh cells; Jonathan C. Aster (Harvard Medical School, Cambridge, MA, USA) for the MigR-GFP and MigR-ICN1-GFP constructs; and Clara Sánchez-González for animal care.

## Conflict of interest

The authors declare that the research was conducted in the absence of any commercial or financial relationships that could be construed as a potential conflict of interest.

## Publisher’s note

All claims expressed in this article are solely those of the authors and do not necessarily represent those of their affiliated organizations, or those of the publisher, the editors and the reviewers. Any product that may be evaluated in this article, or claim that may be made by its manufacturer, is not guaranteed or endorsed by the publisher.

## Glossary

**Table:**

ATAC-seq	assay for transposase-accessible chromatin using sequencing
cCRE	candidate *cis*-regulatory element
Cδ	TCRδ gene constant region
cfosp	*Fos* promoter
Cγ	TCRγ gene constant region
ChIP-seq	chromatin immunoprecipitation using sequencing
DAPT	7(B-(35-difluorophenyl)-1-alanyl)-s-phenyl-glycine t-butyl ester
DN	double-negative
DP	double-positive
Eδ	TCRδ gene enhancer
Eγ	TCRγ gene enhancer
EMSA	electrophoretic mobility shift assay
eRNA	enhancer transcripts
H3K4me3	trimethylated lysine 4 of histone H3
H3K27ac	acetylated lysine 27 of histone H3
ICN1	intracellular NOTCH1 domain
IL-7	interleukin-7
IL-7R	interleukin-7 receptor
qChIP	quantitative chromatin immunoprecipitation
qPCR	quantitative polymerase chain reaction
RNA-seq	transcriptome analysis using sequencing
RT–qPCR	quantitative reverse transcription PCR
SEM	standard error of the mean
SP	single-positive
TCR	T-cell receptor
TCRδ	T-cell receptor δ chain
TCRγ	T-cell receptor γ chain
Vδ1p	TRAV1 promoter
