# Supplementary material for: Interleukin-7 receptor signaling is crucial for enhancer-dependent TCRδ germline transcription mediated through STAT5 recruitment
Source: Front Immunol. 2022 Aug 19;13:943510. doi: 10.3389/fimmu.2022.943510 (PMC9437428; doi:10.3389/fimmu.2022.943510)
Supplement: Supplementary file 1 [file DataSheet_1.pdf]

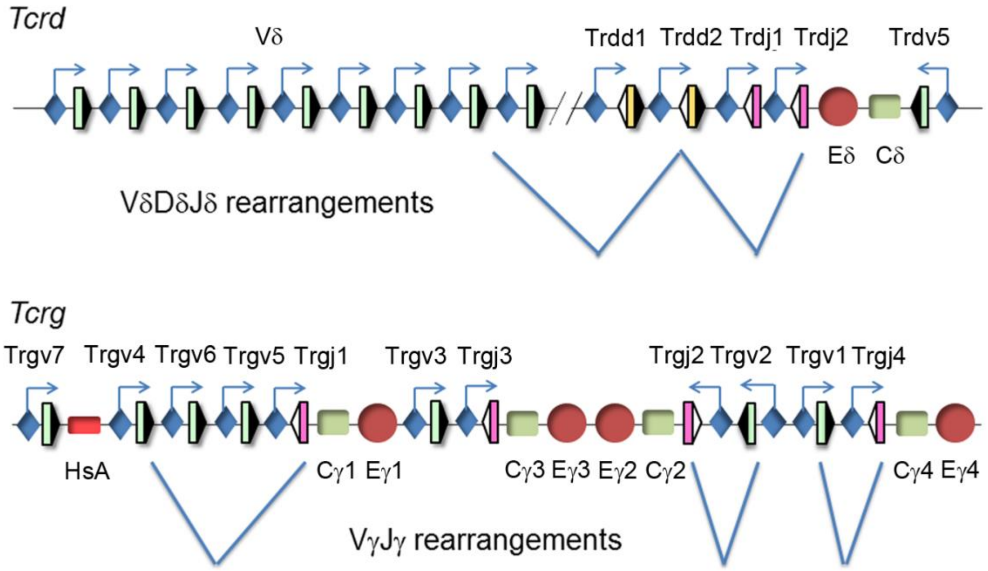

Figure S1. Structure of *Tcrd* and *Tcrg*. Genomic structure of the mouse *Tcra*/*Tcrd* and *Tcrg* loci. V, D and J gene segments are represented by vertical green, yellow and pink rectangles, respectively, and constant regions are represented by green horizontal rectangles. Recombination signal sequences flanking the V, D and J gene segments are represented as black or white triangles, depending on the presence of 23 or 12 base-pair spacers, respectively. Enhancers are represented by red circles, and promoters are represented by blue diamonds. VδDδJδ and VγJγ rearrangement events are indicated by blue lines.

A

TTGAAACCCAGGCCCAGGGCTGGTCCCAGACTGGTTATCTGCAAAGCAAGATTATAACGTGCTCCTGGGA *Trdj2*
CACCCGACAGATGTTTTTTGGAACTGGCATAGAGCTCTTTGTGGAGCCCCGTAAGTTGTTTTTTTTTTTC
TATATTTCTATGGATAATTTGAATCCTGGAACTAGAGCTACCATCCATTTTATATTACAAATTATTACTA
GTAGATTCACTTGCATCTCAGAGTTAGAGTGAGACAGATGTGAAGATGCTGAATTCCTAACATGCTTCTG
TGTACTACTTTTATTTTCAAATATATAGCTATTAAAACTCTAGTCCTTTTTATTTCTTAAATCTACAGAT
CAAGCTAGAAAGTTCAAAGAAATCTTTTTTAAATTACACTAATAAATGTATCACCTAAGGGGAAGGGAGG **EO581863/enhP**
CAGTCATAGGGGGTGCTTGGGCTCAGACTCCCCAGGAAATTGTTCGCTTCCTGCACATACCTTCGGCTGA
GCTAACGTGTCTGCATGCCTCCTTACAGAGCACAAATCTCCTACAGCTCAGAAGGCTTCAGAGACAGGTG
GTTAGCCAAGTCAGCACAACCAGGTAGAACAAACTTCGGGGCTTAAGTGCAATTCCACACCGGCAAAGAG
CCTTGAAATTCCAAGTGACGTTTCTTCTAGATGTCACAATTCTTAGAGAAACAGTAATCCTTCTCTTCTC
TATGTGGTAATGCTCTATGTGGTTCTACCATGGATAATATCTAAGTTTGTAAGTCATAAAGAGCATTTAG
AAAGGATGTTGTCTTCTCTTTGTGTTGGTTCCCAGCAGGTAATTTTAGCATTATATGAGTGCTCATAAGA
GGAGAAGGTCTGAAAACTAAGTGGTATTGTCCAAGCAAATATGCCTGGAAAAGCATTGAAATATAGTTAT
ATTTGTGGAATAACACGAGACTGTTCTCAAAAGATAAACACTGAAATTTTTCACTTAAATCTTATCTTCA
AATATTTGTTTAAATCAAATACAGAATAAACTAAGTTCCATAGGACAACTACATGGAGTACATTCTACTT
ATTTAAAAAGGAAGGTCCAACTTAAGCCAGATTCTGTTTTCTTTAGTTAGCA**TGACAACCAGATGCCAGT**
GAATTGATTGGGTAAACTTTTCCTTCGATTGTTTTAAACATATGGATCTTAACTTAGAGAGAGAGACTGA
GAAAACGTTTTATTAAATCAATGCACTATGTTCAATTTGAAAACCTGACTAATACTTTCAAGGCTCAGCA
TATACATGAATGAA**CCTCTTCAAACATTCATCTTGCC**AGTTATGTTATAATATTCATATTTTCAGAAATT
ATTTCCCTAGCTTAAGATGATTATTGATGATCGCTTCTTAGCGAGTTTAGATTTTAAAAATCACACAAGC
GGCTGGGCCCGGAACCACATACTGTACTATTGGCACTCAGGAAGCTGAGGCAGGAGAATTGAGAGTGTGA
AGTTTGCTTTAACTACTTAGTGAGACCCTGTCTCAGCAAAAATCCAACTTACTCAGACTGGATCATACTC
CTGAGTAACCGATAAGGTAGGGGTTTTGAGTTGGCTTTGGTTAGTTTGGTGGGCTTGTTTTGTTTTGTTT **EO581864/enhP-Eδ**
TGTTTTGTTTGAACAAATTTCTAACTCCTAACTCTCTTGAAAAATCAACCGCAAAATACATGCCTAGCCA
CAAACAAGATAATGAACAAGAGATAGCAGGGCTTCTGAAAGGAGAGGCAAGTGATGAAGACTTGTGGTTT
CCAGCCGTTAATGCTGGGGTTATCAGTTTTGCTGGCAGCAGCTTCAGTGTGTAAGAAGACTAAAAACCTA
AGGTTTTCTCAGGAGGGTTGAGAGCAGATAA**TTCTAAGAA**ACCCTTTGTAAAAAGAAAGAAAGAAAAGAA
AGCCAATCTTGTCGAAAGCCAAAACAGAATCTTAGGAATTAGCCTTCTGCCCTGAGTACCAGAACAGATC
CCAATCTTTCAGTTGAGCT**TCTCGGTGGAGAAAAACTGG**AGAATTATAATGTACTCATGAGGAAGCC**TTC** **EO581865/enhD**
TCTGAACAGCTTGTTCTCTTAGGATATCCCTGATCTGCCCTGATCCAGTTCTACTGTGGAGAGCAGCAGG
CACCTTCTCATCAACAGTCACTCCC**CTCAACCAGTCTGGCCAACT**GATTTGGTTATAGTTTCACTATCAT
GGACCCCATCACTAAAACTCAAACCTAGACTCTGGATAAAGCATTTAAAACATCAATCTGCTTCTTCTTG
CATGTACATTACCAAGATTTTCTGTTAAGGAAGAACCTGCTACCTTGACCATAAACCATAAGAGATAAGC

B

CAGGTTCTCTGGAAAGGAAGCAGAGCGCCATTTTGGAACAACTCATGAACAAGACTCGGAGATAACTCCT
TGATGGTCCTGAGTTGATGCAATGTTTTCCATCCAAGTTCCAGGCCCAGCTTTGCAGACATGGAACTCTT
TCTGCAGTGCCAAGTACAGACACAGAACGGACAAGCAGCAGCAAGAGCTTTGTGAGGCCGTGACCAGCCA
GGATGCAGGCAGCATCTCCTGTGGTCAG**ACATCTCAGCAGCCTCCTTC**CAGAGCCCACCTGGGCTCCCAA **Eγ4**
GCCTCAGCAGTACAGAGTGTCAGTTGCAGGAGAGGAGGGGACACCGGAGGTGTGCAGGATGGCGTTGCTA
TGGTTGGTGGCTTTATTTTCTCAGGCATCTTTCGTTTATCTGTTTGGTCAATGTGGTTGC**TTCCTGGAA**A
ATGCTCAAAGTCAAACCCACCACTACTGACTGGGGCGACACAAAGGGTAGCTAGGGTATTAGAAGAGAAA
ATAAGAGCAGTTAAACCGCAGCCAGCTCTGCTCTGCTTCAAAAGGTCACGGCTAGTAGGAAAAAAAAAAT
TAGCTTCATGCTGTGTGG**TGGCTTCAAACTGCAGAGTC**ACACTGCGGACAGCAGGACTCCTGGGGTGGAC
CCTCGGTGCTCAGCTGCTTCAGCTGGCATCAGATGCTGACACTTTTAACTTGTTTTCTCAGAGTACAAAG
AGGGTGCTAAATGT**GCATTGTGACTCCAGCTGAT**GGCGATGCGGGCCACAGTAATGTGCTTTATGAGCAA
AGCAATGGAAGCCAATCCAATGCACTGAAACTCTCATCTGGGCTGATCCCGGAGGCACCACAGTAACACT
AACTCTAGGATACCATCATGCAGTCAGTAATGAGAGGTCAGAGGACAGGCTGGGTGCAGAGTCGACACCC **EO552802/enhD**
ACACCCCAGGGCTCTTTCGTCCTGACTGCCTCTGAGATCATCTCCCTAGGACGCAGTGTCTGATTTCACA
ATTGCACACATATGATGTGCACCTGTGTCTGTGACCCGTCAACTACACATGGCTGCAGTCTTCCTGAGAG
GTCTGGCTGCATCTGCTGGCTCAGGCTGGCACTGTTTCTCAGCACTGACATCCAACTAGGCTTCAAGTAC
ATACTGCTCCACTCTTGACACAGGCAATTATTCCTGAAATTGTTCCATCAAGTTCCTGAGGTCCTGCCC
CAAGAGCAGACCTAGAGAGTTCCCTCTAGTCTCCCTTACTATAAGTAGCCCTTCTTTGTTGCACCCAGTC **EO552803/enhD**
TCCCTGCCAGACTCCTTGTGGCACTGCTCAAGTGGGCATACTCTTGAAACAGTGCCTATGCAGGACTGGA
TCTGCACAGGGCCCGCTTCCACCCAAGCCTGAAGCATTGCTCCTGCATGCATGCAGTTAACCTTCCTAGC
ATGACCCAGCCAATGATGCAGCCTCACAGTCTGCCCTTCTGGGAAGCCGAAATTGAATTTGGAAAAAATG
TCTGGACAAACAGCCGATGACGTAAATTAGTTCATATTAATCCTAAGGAAGTAAACGTGCAGGAAAAGGC **EO552894/enhD**
GGAGCAAAAGCAGCCACAGCCTTTGCAGGTTGTTTGTGTGCAGCACATCAGACCCTAGCGCAGAACAAGA
AGTCCCAAGAGAGTCATACAAAACACAGGCCAAGAGTTTCTATGCACCCAGGCCACACCCTCTTCCTTTT
ATAACCCACCACTCCTGGACAAACATATAATACAGACAACACACAATCATCATTTTGATACGTTGGATTT
ATTATTTTAAAACTGTATAAAGAGCACATGTGCATGAGTTTAAAAACACAAGTGAAATGTAAGGAGTTAA

Figure S2. Enhancers in the vicinity of Eδ and Eγ4. (A) According to the ENCODE Registry of cCREs, Eδ (EO581864/enhP), in green, is flanked by a proximal enhancer, EO581863/enhP, and a distal enhancer, EO581865/enhD, in yellow. EO581863/enhP and Eδ are considered proximal enhancers because they are less than 2 kb from the Trdj2 gene segment, in turquoise, promoter. (B) According to the ENCODE Registry of cCREs, Eγ4, in green, is followed by three distal enhancers, EO552802/enhD, EO552803/enhD, and EO552804/enhD, in yellow. Sequences corresponding to the primers used to detect Eδ and Eγ4 eRNAs are underlined. STAT5 sites are indicated in red. The mouse GRC38/mm10 reference genome was used.

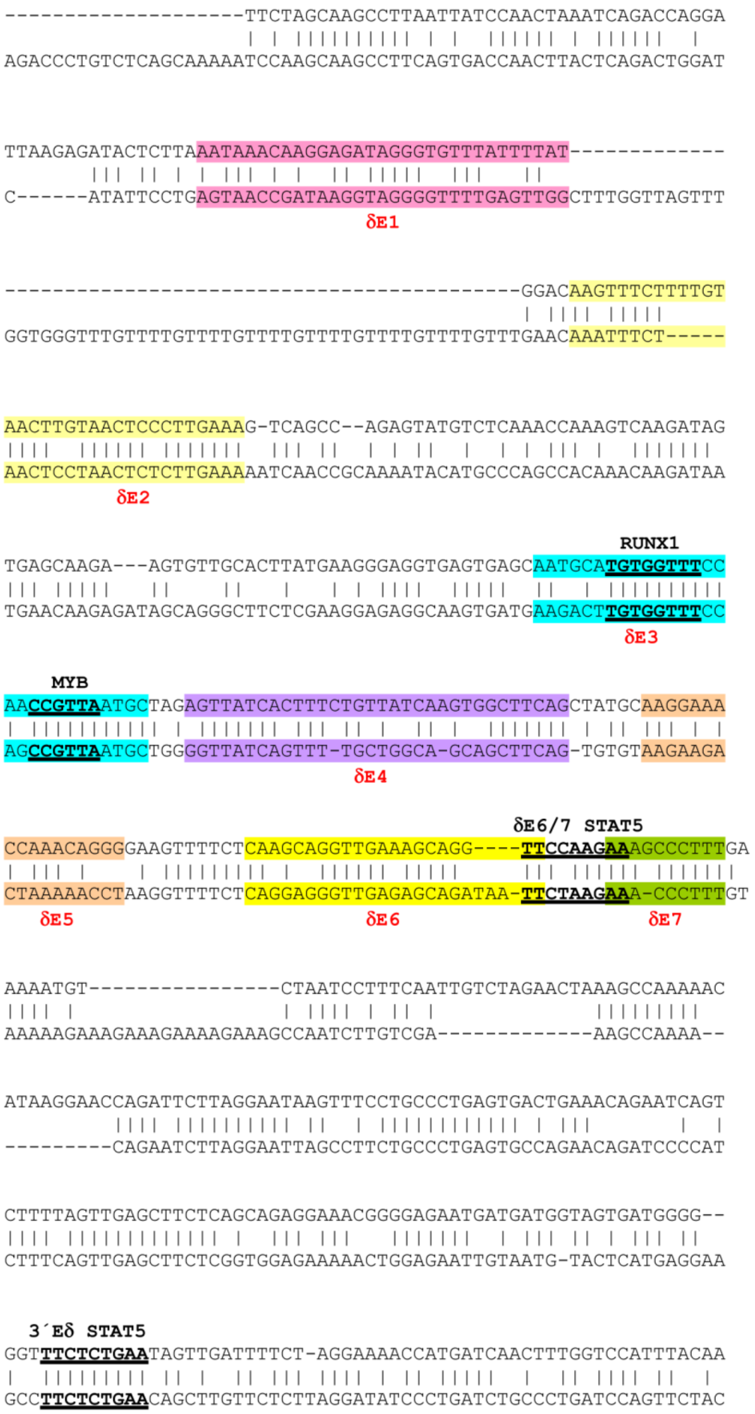

Figure S3. Conserved STAT5-binding sites in Eδ and 3´ region. The Eδ protein binding elements are indicated as follows: δE1 is pink, δE2 is yellow, δE3 is turquoise, δE4 is purple, δE5 is orange, δE6 is bright yellow, and δE7 is green. Essential MYB- and RUNX1-binding sites in δE3 are indicated. Two putative STAT5-binding sites in the human (upper) and mouse (down) Eδ and 3´sequences were found between δE6 and δE7 (δE6/δE7) and the 3´-end of Eδ (3´Eδ). The latter site is located in the distal enhancer EO581865/enhD based on the ENCODE Registry of cCREs and ReMap Atlas of Regulatory Regions.

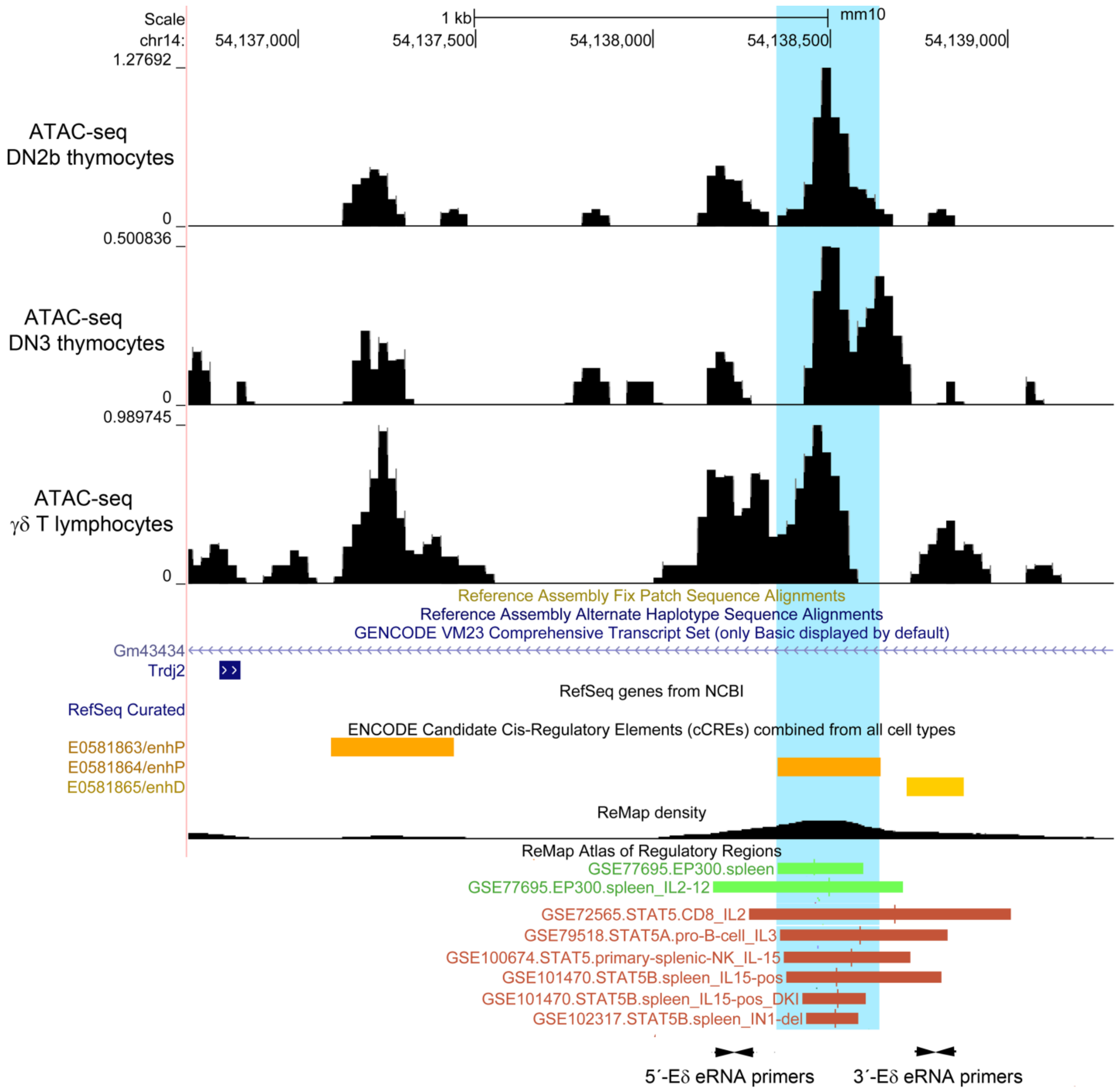

Figure S4. Analyses of chromatin profiles and factor binding to Eδ and surrounding region. Analyses of chromatin accessibility by ATAC-seq in DN2b and DN3 thymocytes and in γδ T lymphocytes (www.immgen.org), transcripts annotated by GENCODE, presence of other *cis*-regulatory regions by the ENCODE Registry of cCREs, and factor binding by ChIP-seq, including p300 and STAT5, using ReMap Atlas of Regulatory Regions in a 2.6 kb region including Eδ are shown. The location of Eδ is highlighted in light blue and the positions of the primers used to detect eRNAs by RT–qPCR are indicated. According to the ENCODE Registry of cCREs, EO581863/enhP and EO581864/enhP (Eδ), shown in orange, are proximal enhancers because they are less than 2 kb from Trdj2 promoter. Eδ is followed by a distal enhancer, EO581865/enhD, shown in yellow. Binding of STAT5 and p300 in immune cells and tissues by ChIP-seq and density profile of factor binding from ReMap are shown. These analyses were mapped to the mouse GRC38/mm10 reference genome using the UCSC browser.

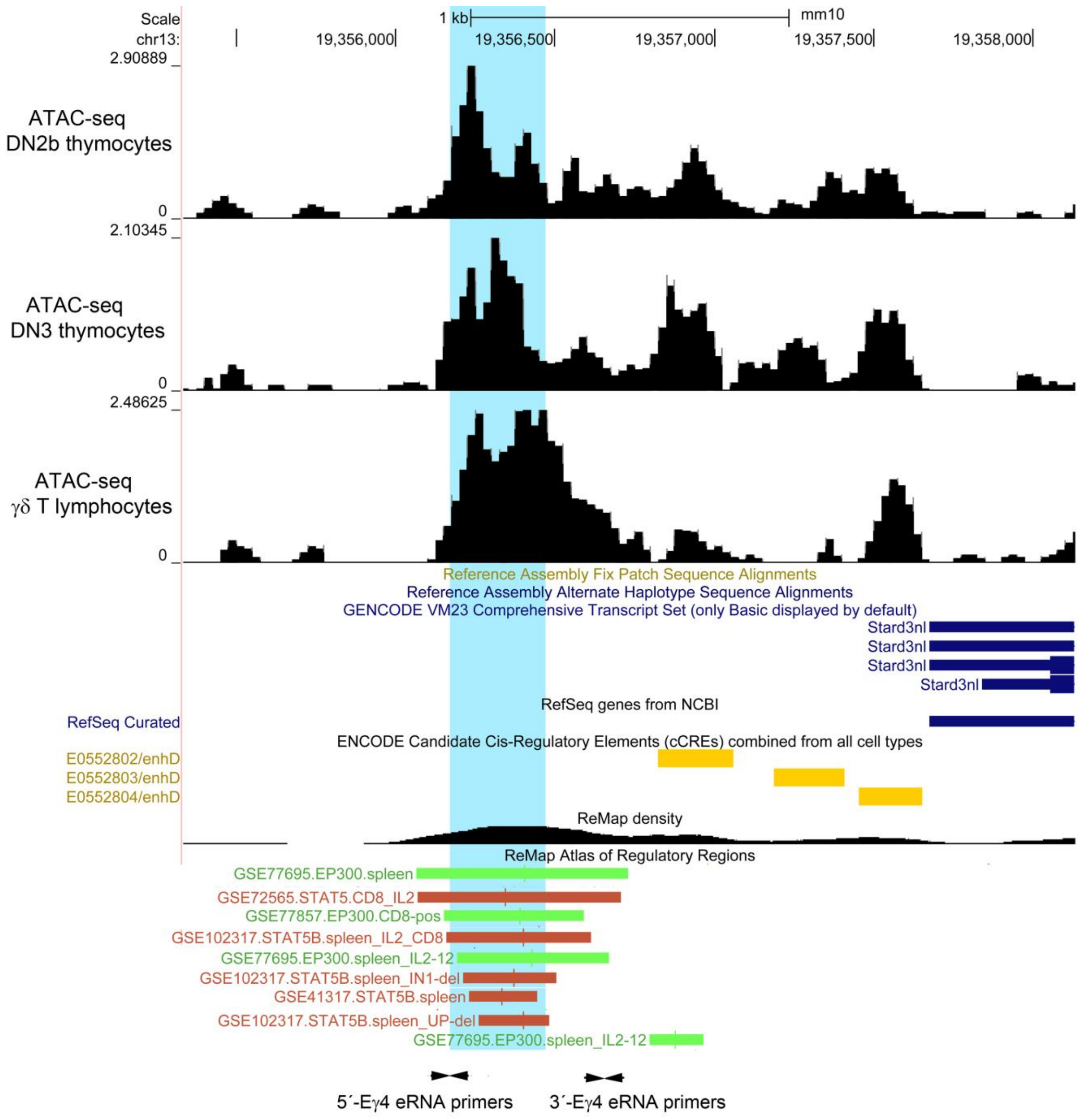

Figure S5. Analyses of chromatin profiles and factor binding to Eγ4 and surrounding region. Analyses of chromatin accessibility by ATAC-seq in DN2b and DN3 thymocytes and in γδ T lymphocytes (www.immgen.org), transcripts annotated by GENCODE, presence of other *cis*-regulatory regions by the ENCODE Registry of cCREs, and factor binding by ChIP-seq, including p300 and STAT5, using ReMap Atlas of Regulatory Regions in a 2.8 kb region including Eγ4 are shown. The location of Eγ4 is highlighted in light blue and the positions of the primers used to detect eRNAs by RT–qPCR are indicated. According to the ENCODE Registry of cCREs, Eγ4 is followed by three distal enhancers, EO552802/enhD, EO552803/enhD, and EO552804/enhD, shown in yellow. Binding of STAT5 and p300 in immune cells and tissues by ChIP-seq and density profile of factor binding from ReMap are shown. These analyses were mapped to the mouse GRC38/mm10 reference genome using the UCSC Browser.

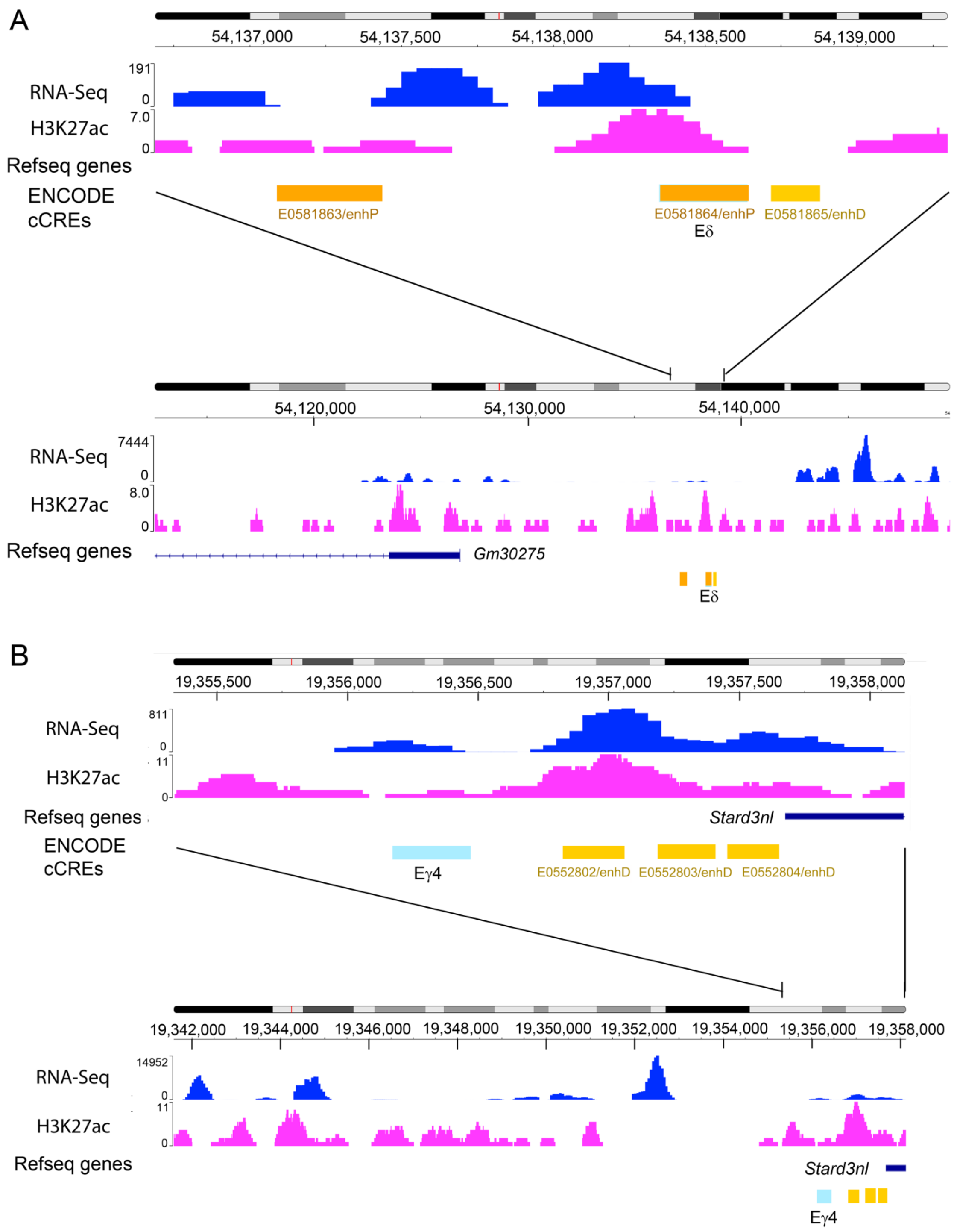

Figure S6. Transcription and detection of H3K27ac at Eδ and Eγ4 and surrounding regions in DN thymocytes. Analyses of transcription by RNA-seq and H3K27ac by ChIP-seq in DN thymocytes (GSE80272) in Eδ (A) and Eγ4 (B) and surrounding regions using the Integrative Genome Viewer (https://igv.org) are shown. The position of proximal and distal enhancers, enhP and enhD, based on the ENCODE Registry of cCREs is indicated. The upper part of each figure corresponds to the regions analyzed in Figures S4 and S5. Analyses were mapped to the mouse GRC38/mm10 reference genome.

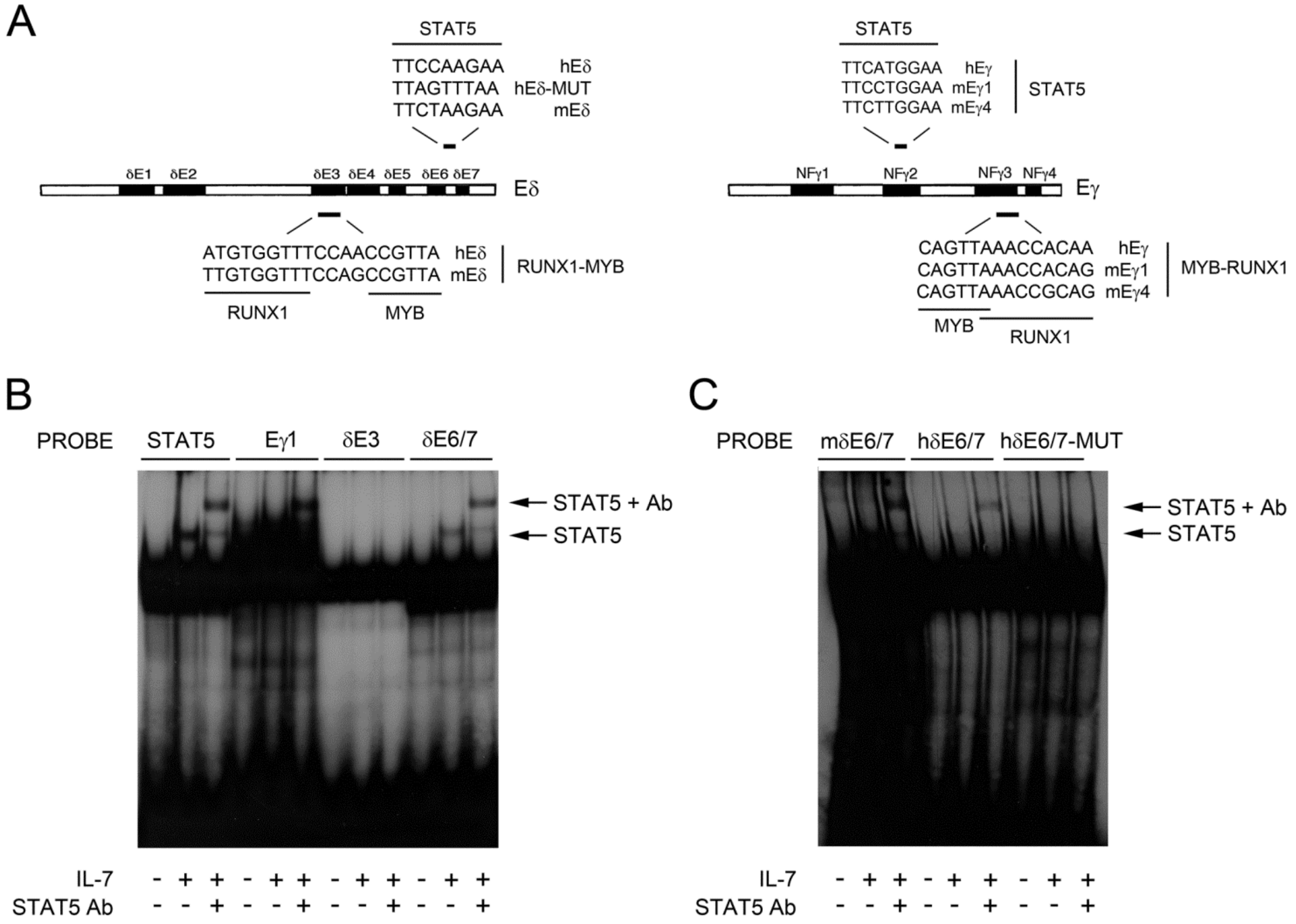

Figure S7. STAT5 binds to a conserved Eδ site. (A) The diagrams represent Eδ and Eγ elements, δE1-δE7 and NFγ1-NFγ4, and depict the location and sequence of the conserved and validated Eδ STAT5-binding site located between δE6 and δE7 (δE6/7) and the sequence of the mutated site (δE6/7-MUT), as well as conserved STAT5-, RUNX1-, and MYB-binding sites in the Eδ and Eγ sequences. **(B)** Analysis of STAT5 binding to a consensus site (STAT5), Eγ1 and δE6/7 as determined by EMSAs. Binding to δE3 was assayed as the negative control. **(C)** Analysis of the STAT5-binding site in mouse δE6/7 (mδE6/7), human δE6/7 (hδE6/7) and mutated human δE6/7 (hδE6/7-MUT) as determined by EMSAs. Radiolabeled probes were incubated with cell extracts obtained from untreated (-) or IL-7-treated (+) SCID.adh cells in the presence or absence of a specific anti-STAT5 antibody (STAT5 Ab). The complexes containing STAT5 and STAT5 + antibody (STAT5 + Ab) are indicated with arrows.

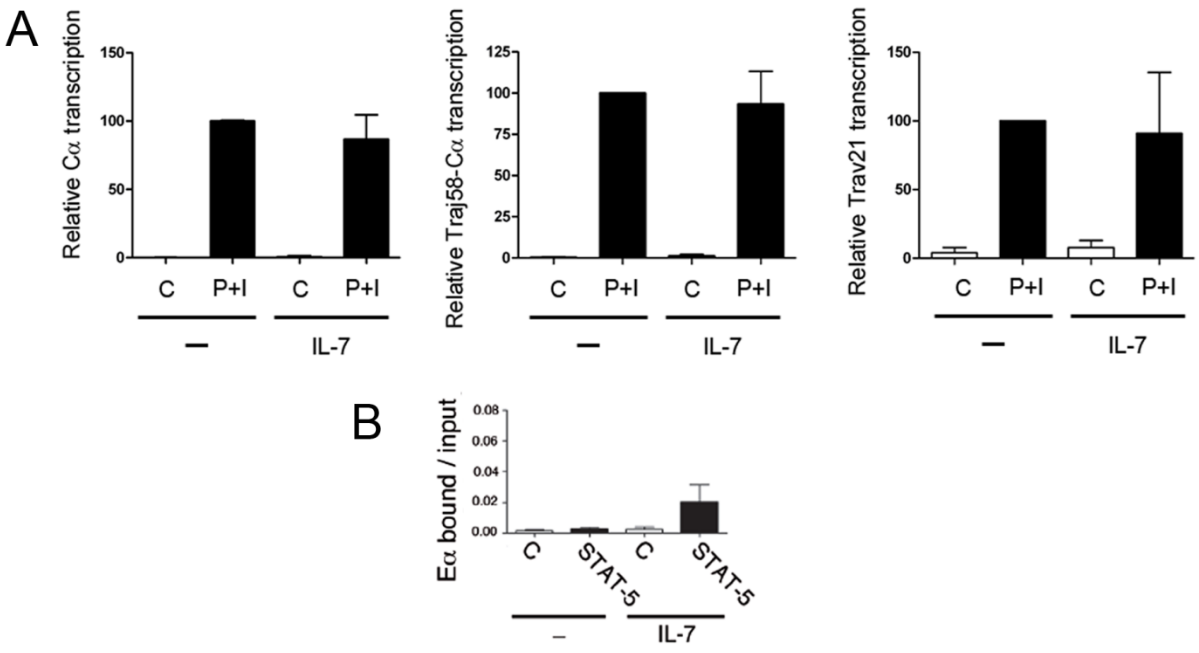

Figure S8. *Tcra* transcription is independent of IL-7R signaling. (A) RT–qPCR analysis of Cα, Traj58-Cα and Trav21 transcription in unstimulated (C) or phorbol-myristate acetate- and ionomycin-stimulated (P+I) SCID.adh cells that were incubated in the absence (-) or presence of IL-7 (IL-7) for 24 hours. The results were normalized to those of *Actb* and represent the mean ± SEM of duplicate RT–qPCRs on the basis of 6 independent experiments. **(B)** Binding of STAT5 to Eα in untreated (-) and IL-7-treated (IL-7) SCID.adh cells after 30 minutes, as determined by qChIP on the basis of 8 independent experiments.

Actb	*qRT-PCR*	5’- ACACCCGCCACCAGTTC	5’- TACAGCCCGGGGAGCAT
**Cδ**	qRT-PCR	5´- TACGACTGCTGTTTGCCAAGA	5´- TGAAGGGTCAGTCTGAAGCA
**Cγ4**	qRT-PCR	5’- CCCAGGAAGGAAACACCATA	5’- TCATGCCGCAAAACACTATC
**Dδ2-Jδ1**	qRT-PCR	5´- GGAGGGATACGAGCACAGTG	5´-TGTCATGGCTAGAGGGCTTT
**Jδ1-Cδ**	qRT-PCR	5´- CCCAAGTGACTGTGGAACCA	5´-TGACAGCACTGTACTTCCCG
**Jδ2-Cδ**	qRT-PCR	5´- TGGAACTGGCATAGAGCTCT	5´-TGACAGCACTGTACTTCCCG
**Eδ eRNA - 5´**	qRT-PCR	5´-TGACAACCAGATGCCAGTGA	5´- GGCAAGATGAATGTTTGAAGAGG
**Eδ eRNA - 3´**	qRT-PCR	5´- TCTCGGTGGAGAAAAACTGG	5´- AGTTGGCCAGACTGGTTGAG
**Eγ4 eRNA – 5´**	qRT-PCR	5’- TGATGGTCCTGAGTTGATGC	5’- GAAGGAGGCTGCTGAGATGT
**Eγ4 eRNA - 3´**	qRT-PCR	5´- TGGCTTCAAACTGCAGAGTC	5´- ATCAGCTGGAGTCACAATGC
**Human *ACTB***	qRT-PCR	5’- CGCGAGAAGATGACCCAGA	5’-TCACGATGCCAGTGGTACG
**Human *IL7RA***	qRT-PCR	5´- GGAGAAAGTGGCTATGCTCAAAA	5´- TCCATTCACTTCCAACTGGCTAT
**Eδ**	ChIP	5´- GAGCTTCTCGGTGGAGAAAA	5´-GACTGGTTGAGGGGAGTGAC
**Eγ4**	ChIP	5’- GACATCTCAGCAGCCTCCTT	5’- TGAGCATTTTCCAAGAAGCA
Oct2	ChIP	5’- CGGGTGTGAGAGGTGTGG	5’- CGAGTCTGAAGCAAGCCAGT
**mEγ1**	EMSA	5´-TAGTTGCTTCCTGGAAAATGGTT	5´-AACCATTTTCCAGGAAGCAACTA
**mδE6/7**	EMSA	5´-CAGATAATTCTAAGAAACCCTTT	5´-AAAGGGTTTCTTAGAATTATCTG
**hδE6/7**	EMSA	5´-AAGCAGGTTCCAAGAAAGCCCTT	5´-AAGGGCTTTCTTGGAACCTGCTT
**STAT5**	EMSA	5´-AAAAGATTTCTAGGAATTCAATC	5´-GATTGAATTCCTAGAAATCTTTT
**STAT5-MUT**	EMSA	5´-AAAAGATTTAGTTTAATTCAATC	5´-GATTGAATTAAACTAAATCTTTT
**hEδ6/7-MUT**	EMSA	5´-AAGCAGGTTAGTTTAAAGCCCTT	5´-AAGGGCTTTAAACTAACCTGCTT
**hEd-STAT5MUT_NEBaseChanger**	Mutagenesis	5´-AAAGCAGGTTAGTTTAAAGCCCTTTG	5´-CAACCTGCTTGAGAAAAC

Table S1. Sequences of the primers used
